# Bridging therapy versus direct endovascular thrombectomy in basilar artery occlusion stroke: a systematic review and meta-analysis

**DOI:** 10.1007/s11357-025-01887-0

**Published:** 2025-09-18

**Authors:** Esra Zhubi, Azamat Bissenov, Marie Anne Engh, Réka Tóth, András Attila Horváth, Peter Hegyi, Bence Gunda

**Affiliations:** 1 Department of Neurology, Semmelweis University, Balassa utca 6, Budapest, H-1083 Hungary; 2 Centre for Translational Medicine, Semmelweis University, Budapest, Hungary; 3 Department of Orthopedics, Semmelweis University, Budapest, Hungary; 4 Neurocognitive Research Center, Nyírő Gyula National Institute of Psychiatry and Addictology, Budapest, Hungary; 5 Research Centre for Natural Sciences, HUN-REN, Budapest, Hungary; 6 Department of Anatomy, Histology and Embryology, Semmelweis University, Budapest, Hungary; 7 Institute for Translational Medicine, Medical School, University of Pécs, Pécs, Hungary; 8 Institute of Pancreatic Diseases, Semmelweis University, Budapest, Hungary

**Keywords:** Ischemic stroke, Meta-analysis, Systematic review, Thrombectomy, Thrombolysis

## Abstract

The benefits and safety of bridging therapy in basilar artery occlusion remain unclear, although current guidelines recommend it based on weak evidence and analogy to anterior circulation strokes. This study compares bridging intravenous thrombolysis and endovascular thrombectomy versus direct endovascular thrombectomy in a larger-than-ever basilar artery occlusion Population. Embase, PubMed, Scopus, Web of Science, and Cochrane Library were systematically searched. Studies that reported 90-day functional independence (mRS: 0–2), 90-day independent ambulation (mRS: 0–3), successful recanalization, symptomatic intracranial hemorrhage, any type of intracranial hemorrhage, and 90-day mortality were included. Two reviewers independently extracted data and assessed bias using ROBINS-I. A random-effects model was used. The protocol was registered in PROSPERO (CRD42024519161). Fifty-eight studies with 9372 subjects were included in the final analysis. Bridging therapy was associated with higher 90-day functional independence (OR 1.46; 95% CI 1.22–1.76; *p* < 0.001) and lower 90-day mortality (OR 0.63; 95% CI 0.49–0.82; *p* = 0.002). No significant differences were found in recanalization rate (OR 0.97; 95% CI 0.79–1.18; *p* = 0.707) or symptomatic intracranial hemorrhage (OR 0.88; 95% CI 0.65–1.18; *p* = 0.330). Benefits were consistent across subgroups defined by stroke severity, treatment window, occlusion site, and study site. Bridging therapy for basilar artery occlusion patients leads to a higher rate of functional independence and lower mortality without increasing the rate of intracranial hemorrhage compared to direct endovascular thrombectomy consistently across all subgroups.

**Supplementary Information:**

The online version contains supplementary material available at 10.1007/s11357-025-01887-0.

## Introduction

Ischemic strokes within the posterior circulation account for approximately 20% of all ischemic strokes [1]. They pose a significant diagnostic and treatment challenge due to their diverse clinical presentation, which can range from transient ischemic attacks of vertigo or minor strokes to advancing brainstem dysfunction or coma at onset, particularly in cases of basilar artery occlusion (BAO) [2]. Strokes from BAO are associated with severe neurological impairments and a potential risk of fatality in almost 80% of strokes. However, they account for only 1–2% of all strokes [3]. Therefore, their optimal management is less studied and requires deeper understanding.

Although several trials have demonstrated significant benefits of endovascular thrombectomy (EVT) on functional outcomes of large vessel occlusions in the anterior circulation [4], the effectiveness of EVT for BAO has been less evident until recently [5]. While earlier randomized controlled trials (RCTs) such as the BEST and BASICS studies have failed to demonstrate clear benefits for EVT [6, 7], recent trials in China, including ATTENTION and BAOCHE, have shown promising results, indicating that EVT within 0–12 h and 6–24 h of symptom onset, respectively, is more effective than medical therapy alone for carefully selected patients with BAO [8, 9].

Bridging EVT with prior intravenous thrombolysis (IVT) may have the potential for improved blood flow restoration by partly dissolving the thrombus and opening up perforating or branching arteries before EVT, which can lead to better neurological recovery [10]. There are, however, significant potential disadvantages, including the risk of clot fragmentation during IVT, which may complicate subsequent EVT procedures and lead to incomplete recanalization [11], increased risk of intracranial hemorrhage, and risk of treatment delay, which could potentially impact overall outcomes [12]. This issue has not been specifically addressed, and current guidelines recommend bridging IVT with EVT in patients with BAO who present within 4.5 h of symptom onset based on weak evidence [13].

The aim of this study is to compare the clinical efficacy and safety of bridging IVT with EVT versus direct EVT in the treatment of acute ischemic stroke due to BAO in a larger-than-ever patient population from different geographical regions.

## Methods

### Study design

The following systematic review and meta-analysis was based on the recommendations of the PRISMA 2020 guidelines [14] and the Cochrane Handbook [15]. The protocol was registered on PROSPERO (CRD42024519161) and adhered to every step, ensuring transparency and minimizing bias.

### Information sources and search strategy

The final systematic search was conducted in five databases: Medline (via PubMed), Embase, Scopus, Web of Science, and Cochrane Central Register of Controlled Trials (Central) in March 2024. Citationchaser [16] was used to identify relevant studies from the reference lists of the eligible articles and cite articles.

### Eligibility criteria

The analysis used the patient, intervention, comparison intervention, and outcome (PICO) model, focusing on comparing the efficacy and safety outcomes of bridging therapy (EVT + IV) and direct EVT in patients with BAO. The primary outcome of interest was 90-day functional independence reported as modified Rankin Scale (mRS) from 0 to 2. Secondary outcomes were 90-day independent ambulation (mRS: 0–3), successful recanalization rate (modified Thrombolysis in Cerebral Infarction (mTICI): 2b-3), symptomatic intracranial hemorrhage, any type of intracranial hemorrhage, and 90-day mortality.

This meta-analysis focused on prospective and retrospective cohort studies, whereas conference abstracts, guidelines, systematic reviews of literature, meta-analyses, animal studies, case reports, and case series were excluded. Studies that reported patients who experienced basilar artery occlusion and underwent treatment with bridging therapy (EVT + IVT) and direct EVT were included. These studies also reported at least one of the outcomes of interest. Studies that reported patients with anterior circulation ischemic strokes, patients with isolated vertebral or posterior cerebral artery stroke, and patients who experienced basilar artery occlusion but did not undergo bridging therapy (EVT + IVT) or direct EVT were excluded.

### Search strategy

We used the following search key, without applying any filters:

(“stroke” OR (“cerebral” AND “infarction”) OR (“brain” AND “infarction”)) AND ((“posterior” AND “circulation”) OR “PCS” OR ((“basilar” AND “artery”) OR “BAO”) AND (“thrombectomy” OR (“endovascular” AND “treatment”) OR “EVT”) AND ((“intravenous “ AND “thrombolysis”) OR “IVT” OR (“thrombolytic” AND “therapy”) OR (“tissue” AND “plasminogen” AND “activator”) OR “rTPA” OR “alteplase” OR (“intravenous” AND “alteplase”)).

### Selection process

The articles retrieved through our search query were imported into reference management software, specifically EndNote 20 [17], by Clarivate Analytics based in Philadelphia, PA, USA, and Rayyan QCRI [18]. Duplicates were automatically and manually removed using the EndNote reference management tool. After duplicate removal, articles were screened by title, abstract, and full text by two independent review authors (EZ, AB) using the Rayyan QCRI. Disagreements between reviewers were resolved by consensus or by the decision of a third independent reviewer (ME). Inter-rater reliability was measured after each phase using Cohen’s kappa coefficient [19].

### Data collection form

Data extraction from eligible articles was performed independently by two authors (EZ, AB) using a Microsoft Excel data collection form (Microsoft Corp. Microsoft Excel 2019, Redmond, WA, USA). The following data were extracted from each eligible article: name of the first author, year of publication, basic demographic characteristics (number of patients, number of female patients, age), number of patients treated with bridging therapy (EVT + IVT) and those with direct EVT, number of events in each group for each outcome, length of follow-up period, and the outcome of interest. Any discrepancies or inquiries during the data extraction process were resolved through discussions between the authors.

### Risk of bias assessment

Two reviewers (EZ and AB) independently evaluated the risk of bias in the studies using the ROBINS I tool. A third investigator (ME) resolved any disagreements.

### Certainty of evidence

The Grading of Recommendations Assessment, Development, and Evaluation (GRADE) approach [20] was used to evaluate the quality of evidence for the outcomes in our clinical question. We used GRADEpro GDT 2015 (software; McMaster University and Evidence Prime, 2022, available from gradepro.org) to interpret the results.

### Statistical analysis

As we assumed considerable between-study heterogeneity, we used random-effect models. The odds ratio (OR) was used as the effect size measure with 95% confidence intervals (CI). Where possible, the total number of patients and those with the event of interest were extracted or calculated (“raw data”). Otherwise, the (adjusted) OR with 95% CI was extracted (assuming a Wald-type interval). Pooled OR based on raw data was calculated by the exact Mantel–Haenszel method (without continuity correction) [21, 22]. The inverse variance weighting method was used for adjusted outcomes and those including studies without raw data. For subgroup analysis, we used a mixed-effects model. To assess the difference between subgroups, we used a “Cochrane *Q*” test at a 5% significance level.

Results were considered statistically significant if the CI did not contain 1. We summarized meta-analysis findings in forest plots, including prediction intervals (i.e., the expected range of effects in future studies). Higgins and Thompson’s *I*^2^ [23] statistics also described between-study heterogeneity. All statistical analyses were conducted with R [24] (R Core Team 2023, v4.3.2) using the meta (Schwarzer 2023, v6.5.0) package for basic meta-analysis calculations and plots, and the dmetar (Cuijpers, Furukawa, and Ebert 2023, v0.0.9000) package [25] for additional influential analysis.

To estimate the heterogeneity variance measure (*τ*2), the Paule-Mandel method was used where all studies had raw data, and the restricted maximum-likelihood estimator otherwise, with the *Q* profile method for CIs. We used the Hartung-Knapp adjustment. Small-study publication bias was assessed by visual inspection of the funnel plot. If there were at least 10 studies per outcome, Peters (modified Egger’s) test was performed for raw data outcomes and classical Egger’s test otherwise, considering possible small study bias with p values below 0.1. Potential outlier publications were explored using different influence measures and plots following the recommendations of Harrer et al. Model fitting parameters and potential outlier publications were explored using different influence measures and plots (e.g., leave-one-out analysis for change in fitted values, Baujat diagnostics values and plot). In the case of 0 cell counts, individual study OR with a 95% CI was calculated by adding 0.5 as a continuity correction. This was used for visualization, and pooling only if there were studies without raw data in the same outcome. One study reported 0.00 as the lower CI for the adjusted OR. To calculate the SE, we took it as 0.001, since all the other values were rounded to 2 decimal places.

### Reporting bias assessment

Funnel plots were applied to report and visualize publication bias.

## Results

### Literature search results

The search of the databases yielded 6274 initially relevant records. Ultimately, 58 eligible studies involving more than 9372 patients were included in the final analysis. In total, 35 studies involved patients with BAO, and 23 studies involved patients with BAO and vertebral artery occlusion. Of these studies, 34 were involved in the analysis of functional independence, 24 studies were involved in the analysis of independent ambulation, 11 studies in the analysis of successful recanalization, 8 studies in the analysis of sICH, 7 studies in the analysis of ICH, and 13 studies in the analysis of 90-day mortality.

The entire selection process is illustrated in the PRISMA diagram in sFigure 1. Our calculation yielded a Cohen’s coefficient (*κ*) of 0.62 and an agreement of 82.35% for full-text selection.

### Basic characteristics of studies included

The final eligible studies were published between 2014 and 2024. Table 1 presents the detailed characteristics of the studies included. Thirty-eight studies were from prospective cohorts, and 20 studies were from retrospective cohorts.

**Table 1:** Basic characteristics of included studies

**Study**	**Study design**	**Study period**	**Study site**	**No. of patients ‡**	**No. of females (%)**	**Age ‡**	**Outcomes**	**NIHSS at admission ‡**	**EVT treatment window**	**IVT administration**
Basilar artery occlusion										
Abbas et al. 2023 [26]	Retrospective cohort	Jan/14–Mar/22	USA	74	33 (44.6%)	62.7 (± 16.6)	Independent ambulation; Successful recanalization	16 (9–26)	Not specified	Alteplase within 4.5 h
Baek et al. 2014 [27]	Retrospective cohort	Dec/10–Dec/12	South Korea	25	11 (44%)	68	Functional independence	11 (3–25)	0–8 h	Alteplase (0.9 mg/kg) within 4.5 h
Brissette et al. 2024 [28]	Retrospective cohort	Jan/12–Dec/19	Canada, Ireland, Belgium (6 centers)	279	109 (39.1)	65 (8–96)	Independet ambulation	Not specified	0–24 h	Not specified
Cao et al. 2021 [29]	Retrospective cohort	Jan/13–Sep/19	China	101	18 (18.6%)	62.2 (± 12.91)	Functional independence; Mortality	30 (22.5–36.5)	0–24 h	Not specified
Carneiro et al. 2015 [30]	Retrospective cohort	Jan/12–Dec/14	Portugal	24	7 (29%)	57 (± 14)	Functional independence	23 (8)	Not specified	Alteplase (0.9 mg/kg) within 4.5 h
Dornak et al. 2015 [31]	Retrospective cohort	Jan/06–Nov/13	Czech Republic (multicenter)	72	21 (29.2%)	59.1 (± 13.3)	Independent ambulation	20.8 (± 8.8)	Not specified	Not specified
Feil et al. 2023 [32]	Prospective cohort	Jun/15–Dec/19	Germany	640	277 (43.3%)	72.2 (± 13.3)	Functional independence; Succesful recanalization; Mortality	17 (8–27)	Not specified	Alteplase (0.9 mg/kg)
Giorgianni et al. 2018 [33]	Retrospective cohort	Jan/10–Dec/15	Italy (12 centers)	102	37 (36.3%)	68 (57–76)	Functional independence	17	0–24 h	Alteplase (0.9 mg/kg) within 4.5 h
Gory et al. 2018 [34]	Prospective cohort	Mar/10–Apr/17	France (3 centers)	117	70 (59.8%)	67.7 (12.9) vs 62.9 (16.2) (dead vs alive)	Mortality	22 (14–41) vs 12 (8–21) (dead vs alive)	0–12 h	Not specified
Guenego et al. 2021 [35]	Prospective cohort	Jan/12–May/19	France (multicenter)	50	17 (34%)	63 (54–75)	Functional independence	≤ 6	Not specified	Not specified
Hu et al. 2017 [36]	Retrospective cohort	Jan/13–Aug/16	South Korea	24	11 (45.8%)	65.7 (32–85)	Functional independence; Succesful recanalization	13.9 (2–21) vs 15.3 (6–34) (successful vs not successful recanalisation)	Not specified	Alteplase (0.9 mg/kg) within 4.5 h
Kaneko et al. 2021 [37]	Retrospective cohort	Jan/15–Mar/19	Japan (12 centers)	73	26 (35.6%)	77 (68–84)	Functional independence	24 (13–30)	Not specified	Alteplase (0.6 mg/kg) within 4.5 h
Kang et al. 2018 [38]	Retrospective cohort	Jan/11–Aug/17	South Korea	212	92 (43.4%)	71 (64–78)	Functional independence	17 (10–23.75)	0–12 h	Alteplase (0.9 mg/kg) within 4.5 h
Karadeli 2023 [39]	Retrospective cohort	2016–2021	Turkey	22	10 (45.5%)	61.7 (± 11.3)	Functional independence; Mortality	20 (6–28) vs 18 (8–25) (dead vs alive)	Not specified	Alteplase (0.9 mg/kg) within 4.5 h
Karamchandani et al. 2021 [40]	Retrospective cohort	Jan/17–Jan/20	USA	65	28 (43%)	67 (57–77)	Functional independence	16 (6–28)	0–24 h	Not specified
Kim et al. 2019 [41]	Prospective cohort	Jan/12–Jan/18	South Korea	45	13 (29.9%)	69 (57–78)	Functional independence	16.5 (± 8.4)	0–24 h	Not specified
Lee et al. 2018 [42]	Prospective cohort	Jan/10–Mar/17	South Korea	194	78 (40.2%)	68.8 (± 11.8)(21–92)	Functional independence	16 (7–25)	0–24 h	Alteplase (0.6–0.9 mg/kg) within 4.5 h
Li et al. 2018 [43]	Prospective cohort	Jan/14–Dec/16	China	68	9 (13.2%)	57.9 (± 11.8)	Independent ambulation	24.5 (15–30)	0–24 h	Not specified
Liu et al. 2023 [44]	Retrospective cohort	Dec/19–Jul/21	China	55	11 (20%)	68 (60–75)	Independent ambulation	20 (9–35)	0–24 h	Not specified
Liu et al. 2023 [45]	Retrospective cohort	Jan/12–Dec/18	China	116	20 (17.2%)	59.1 (± 11.7)	Independent ambulation	19 (12–26)	Not specified	Not specified
Mierzwa et al. 2024 [46]	Retrospective cohort	Jan/15–Dec/21	USA	444	198 (44.5%)	66 (± 15)	Independent ambulation; sICH; any ICH	13 (6–24) vs 21 (12–28) (good vs poor clinical outcome)	0–24 h	Not specified
Mourand et al. 2014 [47]	Prospective cohort	Nov/09–Mar/11	France	31	16 (52%)	61.2 (16.9)	Functional independence	14 (7–38)	0–24 h	Alteplase (0.9 mg/kg)
Nappini et al. 2021 [48]	Prospective cohort	2011–2017	Italy	464	161 (35%)	67.7 (± 13.28)	Functional independence; Mortality	18 (10–30)	0–12 h	Alteplase (0.9 mg/kg) within 4.5 h
Neuberger et al. 2019 [49]	Prospective cohort	Jan/12–Sep/17	Germany	101	42 (41.5%)	70.3 ± 13.4 vs 72.5 ± 12.4 (ICH vs no ICH)	sICH; any ICH	30 (20–37.5) vs 26 (11–35) (ICH vs no ICH)	Not specified	Alteplase (0.9 mg/kg) within 4.5 h
Ouyang et al. 2022 [50]	Retrospective cohort	Jan/18–Jun/21	China	55	8 (14.6%)	64 (57–75)	Functional independence	35 (20–35)	0–24 h	Not specified
Pasarikovski et al. 2020 [51]	Prospective cohort	Jan/13–Mar/19	Canada	43	17 (39%)	67 (57–97)	Independent ambulation	18 (6–28)	> 24 h	Alteplase (0.9 mg/kg) within 4.5 h
Pop et al. 2023 [52]	Prospective cohort	Jan/14–May/19	France (18 centers)	195	77 (39.5%)	65 (16)	Independent ambulation	17 (22)	0–8 h	Not specified
Ramazanoglu et al. 2023 [53]	Retrospective cohort	Jan/18–Mar/21	Turkey	57	21 (36.8%)	64.1 (± 14.5)	Mortality	16 (2–26)	Not specified	Alteplase (0.9 mg/kg) within 4.5 h
Ritvonen et al. 2021 [54]	Retrospective cohort	Jun/95–Dec/19	Finland	103	40 (38.8%)	73 (63–79)	Independent ambulation	Not specified	Not specified	Not specified
Ryu et al. 2023 [55]	Retrospective cohort	Jan/12–Jul/22	South Korea	42	19 (45.2%)	70.3 (± 11.2)	Independent ambulation	17 (12–24) vs. 8 (6–15)	0–24 h	Not specified
Singer et al. 2015 [56]	Retrospective cohort	Jan/11–Jun/13	Germany	148	52 (35%)	71 (61–77)	Functional independence	20 (9–28)	Not specified	Not specified
Siow et al. 2022 [57]	Prospective cohort	Jan/15–Dec/19	Belgium, Germany, Greece, UK, Sweden, Singapore, Taiwan	322	116 (36%)	67.5 (± 14.1)	Functional independence; Independent ambulation; sICH; Succesful recanalization	16 (8–25)	0–24 h	Alteplase (0.9 mg/kg) within 4.5 h
Son et al. 2016 [58]	Retrospective cohort	Mar/11–Dec/14	South Korea	19	9 (47.3%)	65.7 (± 9.3)/68 (47–83)	Functional independence	17.9 (± 8.9)/14 (5–34)	0–24 h	Alteplase (0.9 mg/kg) within 3 h onset (before January 2014) 4.5 h (after January 2014)
Uno et al. 2017 [59]	Retrospective cohort	Oct/11–Sep/16	Japan	34	11 (32%)	72 (66–77)	Functional independence	29 (14–33)	0–8 h	Alteplase (0.6 mg/kg) within 4.5 h
Yoon et al. 2015 [60]	Retrospective cohort	Dec/10–Feb/15	South Korea	50	24 (48%)	71 (63–77)	Functional independence	10.5 (7.75–16.00)	0–12 h	Not specified
Vertebro-basilar artery occlusion										
Abdelrady et al. 2023 [61]	Prospective cohort	Jan/15–Dec/19	France	139	48 (35%)	69 (61–76)	Functional independence; Succesful recanalization; Mortality	15 (9–24)	Not specified	Not specified
Alexandre et al. 2021 [62]	Retrospective cohort	Jan/16–Jul/19	France, Switzerland, Italy (10 centers)	191	61 (31.9%)	68.3 (± 13.97)	Functional independence; Succesful recanalization	12 (7–20)	Not specified	Alteplase (0.9 mg/kg) within 4.5 h
Chen et al. 2022 [63]	Prospective cohort	Jan/14–May/19	China (47 centers)	644	163 (25.31%)	64 (56–73)	Independent ambulation	27 (17–33)	0–24 h	Alteplase (0.9 mg/kg) within 4.5 h
Guo et al. 2024 [64]	Retrospective cohort	Jan/14–May/19	China	647	164 (25.3%)	64 (56–73)	Functional independence; Independent ambulation; Mortality; sICH; Succesful recanalization	27 (17–33)	0–24 h	Alteplase (0.9 mg/kg) within 4.5 h or urokinase (1~1.5 million international units) within 6 h of onset
Hirai et al. 2023 [65]	Retrospective cohort	Dec/13–Feb/21	Japan (multicenter)	86	33 (38.4%)	73.5 (67–81)	Independent ambulation	21 (9–32)	0–24 h	Alteplase (0.9 mg/kg) within 4.5 h
Huang et al. 2022 [66]	Retrospective cohort	Dec/15–Dec/18	China (21 centers)	508	147 (28.4%)	61.4 (± 14.5)	Functional independence; Independent ambulation	15 (10–23)	0–24 h	Alteplase (0.9 mg/kg) within 4.5 h
Ishiwada et al. 2023 [67]	Retrospective cohort	Dec/13–Feb/21	Japan (multicenter)	100	34 (34%)	73 (66–82)	Independent ambulation	20 (9–33)	Not specified	Alteplase (0.6 mg/kg) within 4.5 h
Jiang et al. 2021 [68]	Retrospective cohort	Jan/12–Dec/17	China	67	23 (34.3)	63(57–68)	Independent ambulation	13(10–22)	0–24 h	Alteplase (0.9 mg/kg) within 4.5 h
Lee et al. 2020 [69]	Retrospective cohort	Jan/11–Feb/16	South Korea	71	30 (42%)	67 (± 11)	Functional independence	16.5 (9–22.25) vs 22.0 (15.0–27.5) (good vs poor)	0–24 h	Not specified
Lee et al. 2020 [70]	Retrospective cohort	Mar/10–Dec/17	South Korea	40	9 (22.5%)	66.4 (± 9.24) vs 67.1 (± 13.2) (good vs poor clinical outcome)	Functional independence	10.0 (± 4.64) vs 14.8 (± 13.2) (good vs poor outcome)	0–24 h	Alteplase within 4.5 h
Liao et al. 2023 [71]	Prospective cohort	Jan/14–May/19	China (47 centers)	585	143 (24.4%)	64 (56–73)	Independent ambulation	27 (17–33)	0–24 h	Alteplase (0.9 mg/kg) within 4.5 h
Liu et al. 2021 [72]	Retrospective cohort	Jun/12–Mar/18	China	107	25 (14%)	60 (52–68)	Independent ambulation	20 (12–27)	0–24 h	Alteplase (0.9 mg/kg) within 4.5 h
Maier et al. 2023 [73]	Prospective cohort	Jan/15–Dec/21	France (21 centers)	246	85 (34.5%)	66.7 (16.0) vs 66.8 (15.0) (bridging vs no bridging)	Functional independence; Independent ambulation; Mortality; sICH; any ICH; Succesful recanalization	14 (14) vs 13 (11)	0–24 h	Alteplase (0.9 mg/kg) within 4.5 h
Nie et al. 2022 [74]	Prospective cohort	Jul/18–Oct/20	China	310	70 (22.58%)	61.39 ± 10.92	Functional independence; Mortality; sICH; ICH; Succesful recanalization	21 (11–27)	0–24 h	Alteplase (0.9 mg/kg) within 4.5 h
Rentzos et al. 2018 [75]	Retrospective cohort	Jan/91–Dec/15	Sweden	110	36 (33%)	62 (± 13)	Functional independence	31 (13–31)	0–24 h	Alteplase (0.9 mg/kg) within 3 h onset (before 2008) 4.5 h (after 2008)
Sang et al. 2019 [76]	Prospective cohort	Jan/16–Jul/18	China	48	12 (25%)	70.5 (62–80)	Independent ambulation	22 (12.5–26)	0–24 h	Not specified
Sun et al. 2019 [77]	Retrospective cohort	Jan/12–Jul/18	China	187	30 (16%)	60 (± 10)	Functional independence; Independent ambulation; sICH; any ICH; Mortality; Succesful recanalization	22 (10–34)	Not specified	Alteplase (0.9 mg/kg) within 4.5 h
Sun 2023 [78]	Retrospective cohort	Jul/20–Nov/21	China	65	10 (15.4%)	64(55–68)	Independent ambulation	17(12–27)	0–24 h	Alteplase (0.9 mg/kg) within 4.5 h
Sun et al. 2023 [79]	Prospective cohort	Nov/17–Mar/19	China	347	72 (20.7%)	64 (54–72)	Independent ambulation	22 (11–35)	0–24 h	Not specified
Werner et al. 2016 [80]	Retrospective cohort	Nov/08–Jul/13	Spain	28	13 (31.8%)	60.5 (50–75)	Functional independence; Mortality	24 (11.5–31.25)	0–24 h	Not specified
Wu et al. 2021 [3]	Prospective cohort	Dec/12–Dec/18	China	177	33 (18.6%)	59.7 (± 11.8)	Independent ambulation	22 (14–32)	0–24 h	Alteplase (0.9 mg/kg) within 4.5 h
Wu et al. 2021 [81]	Retrospective cohort	Jan/14–Dec/19	China	100	23 (23%)	62 (± 11)	Mortality	26 (17–29)	0–24 h	Not specified
Zhang et al. 2019 [82]	Retrospective cohort	Apr/12–Feb/18	China	103	14 (13.6%)	58.56 (± 9.08)	Functional independence	20.26 ± 10.15	0–24 h	Alteplase within 4.5 h

^**‡**^Parameters represented as mean with standard deviation, or median with range (minimum and maximum)

*EVT* endovascular thrombectomy, *ICH* intracerebral hemorrhgae, *IVT* intravenous thrombolysis, *NIHSS* National Institute of Health Stroke Scale, *sICH* symptomatic intracerebral hemorrhage

### Functional independence (90-day mRS 0–2)

**Fig. 1:**
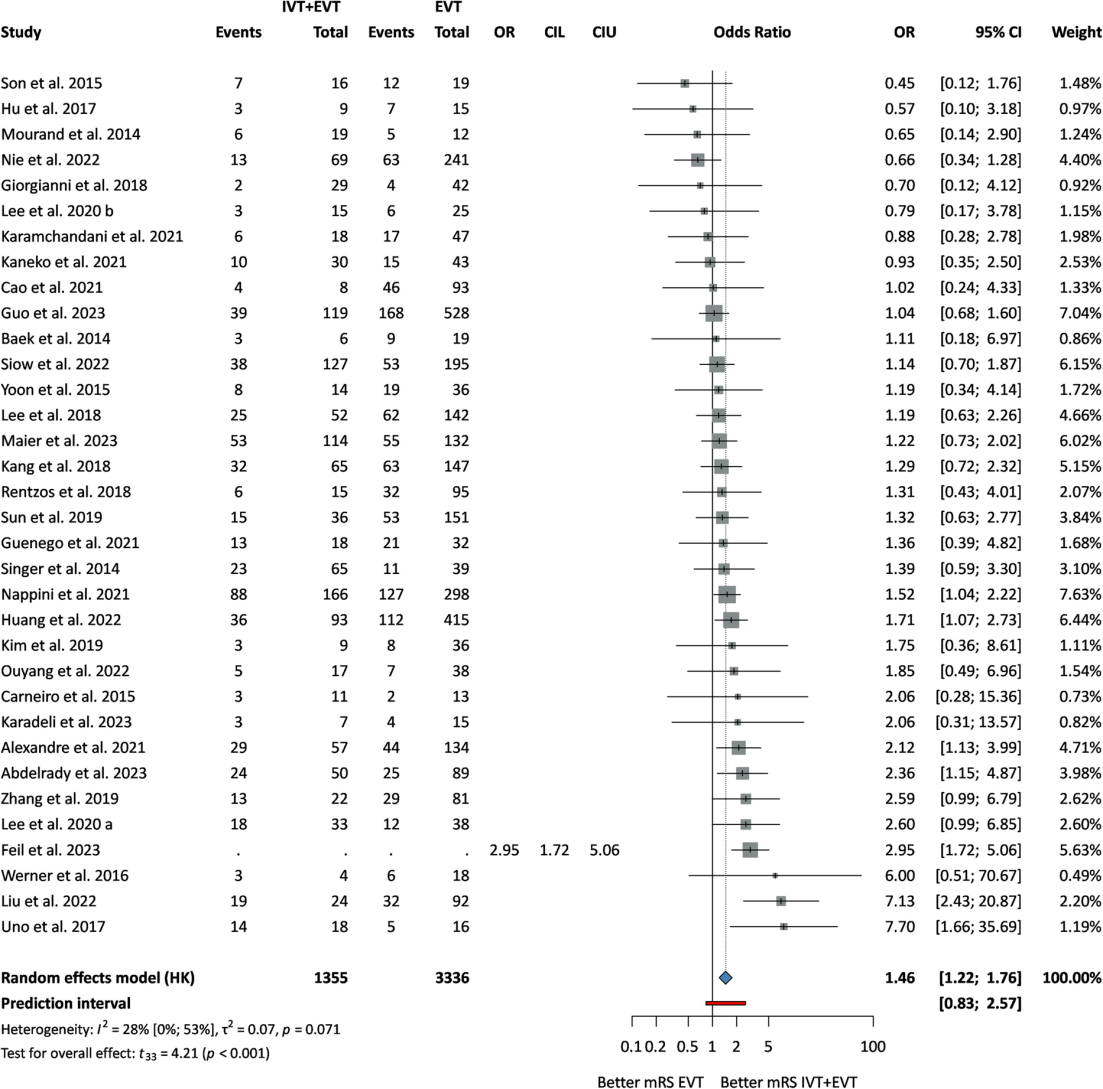
Forest plot representing the odds ratio (OR) of 90-day functional independence (modified Rankin Scale 0–2)

Thirty-four studies reported 90-day functional independence among more than 1355 patients treated with bridging therapy and 3,336 patients treated with direct EVT. Functional independence was defined across studies as a 90-day mRS from 0 to 2. Patients who underwent bridging therapy were more Likely to have functional independence at 90days (OR, 1.46; 95% CI, 1.22–1.76; *p* < 0.001). There was a low heterogeneity among the included studies (*p* for heterogeneity = 0.071; *I*^2^ = 28%) (Fig. 1).

### Independent ambulation (90-day mRS 0–3)

**Fig. 2:**
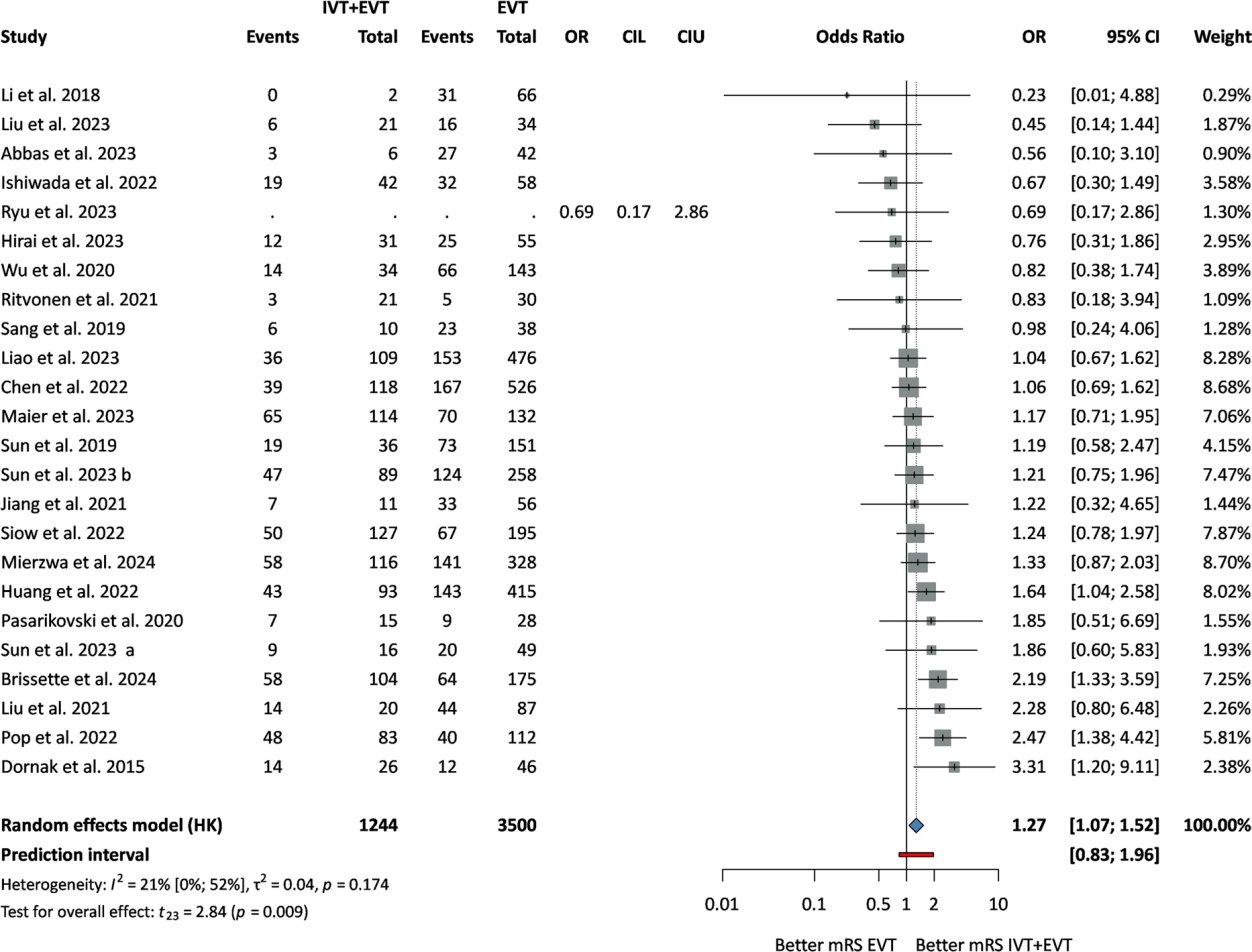
Forest plot representing the odds ratio (OR) of 90-day independent ambulation (modified Rankin Scale 0–3)

Twenty-four studies reported 90-day independent ambulation among more than 1244 patients treated with bridging therapy and 3500 patients treated with direct EVT. Independent ambulation was defined across studies as a 90-day mRS from 0 to 3. Patients who underwent bridging therapy were more likely to have independent ambulation (OR, 1.27; 95% CI, 1.07–1.52; *p* = 0.009). There was a low heterogeneity among the included studies (*p* for heterogeneity = 0.174; *I*^2^ = 21%) (Fig. 2).

### Successful recanalization rate

**Fig. 3:**
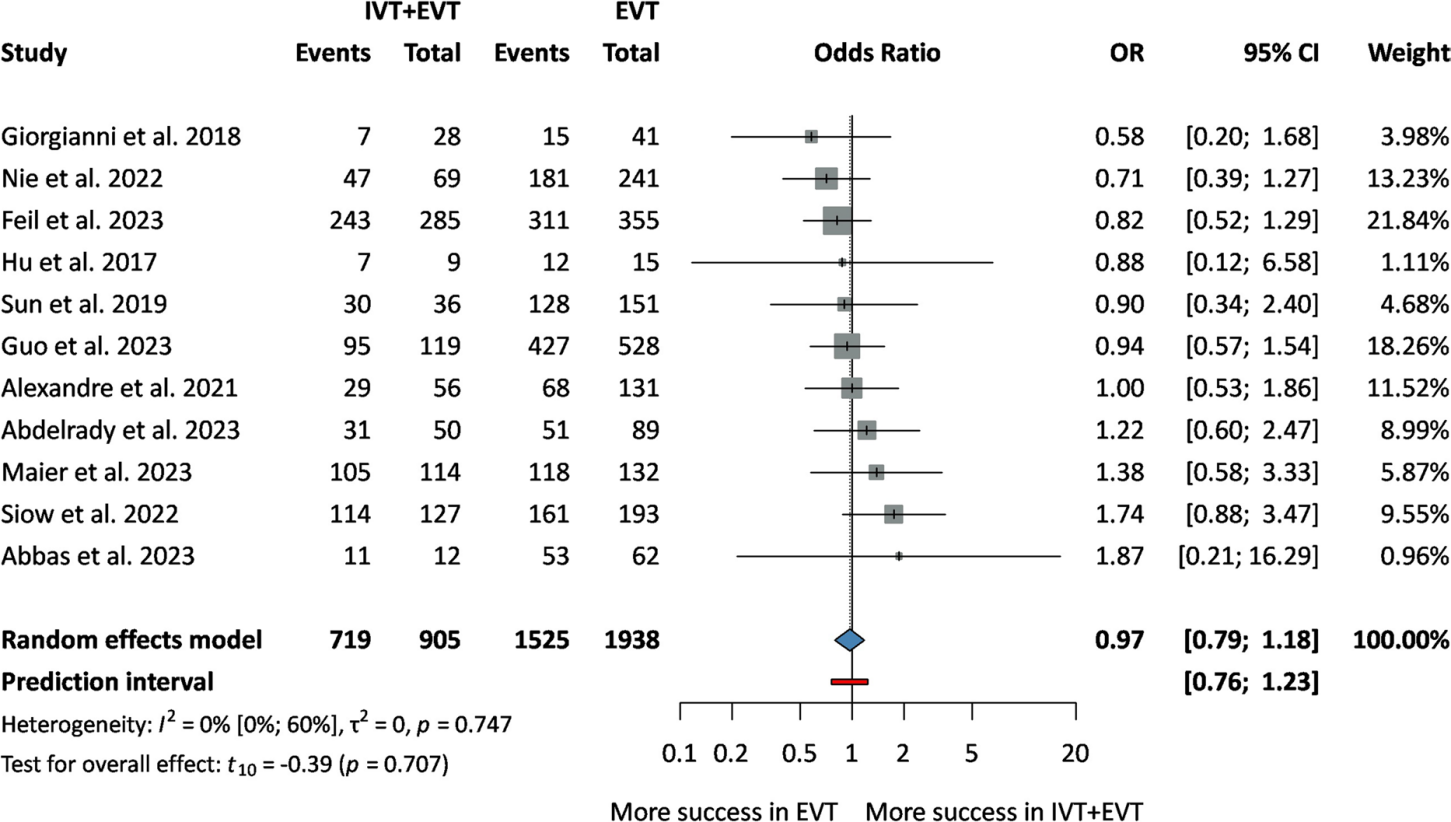
Forest plot representing the odds ratio (OR) of successful recanalization

Eleven studies reported successful recanalization rates among 905 patients who underwent bridging therapy and 1938 patients who underwent direct EVT. Successful recanalization was defined across studies as a mTICI of 2b (near-complete recanalization) to 3 (complete recanalization). There were no significant differences between patients who underwent bridging therapy and those who underwent direct EVT in terms of successful recanalization rate (OR, 0.97; 95% CI, 0.79–1.18; *p* = 0.707). There was a low heterogeneity among the included studies (*p* for heterogeneity = 0.747; *I*^2^ = 0%) (Fig. 3).

### Symptomatic intracranial hemorrhage (sICH)

**Fig. 4:**
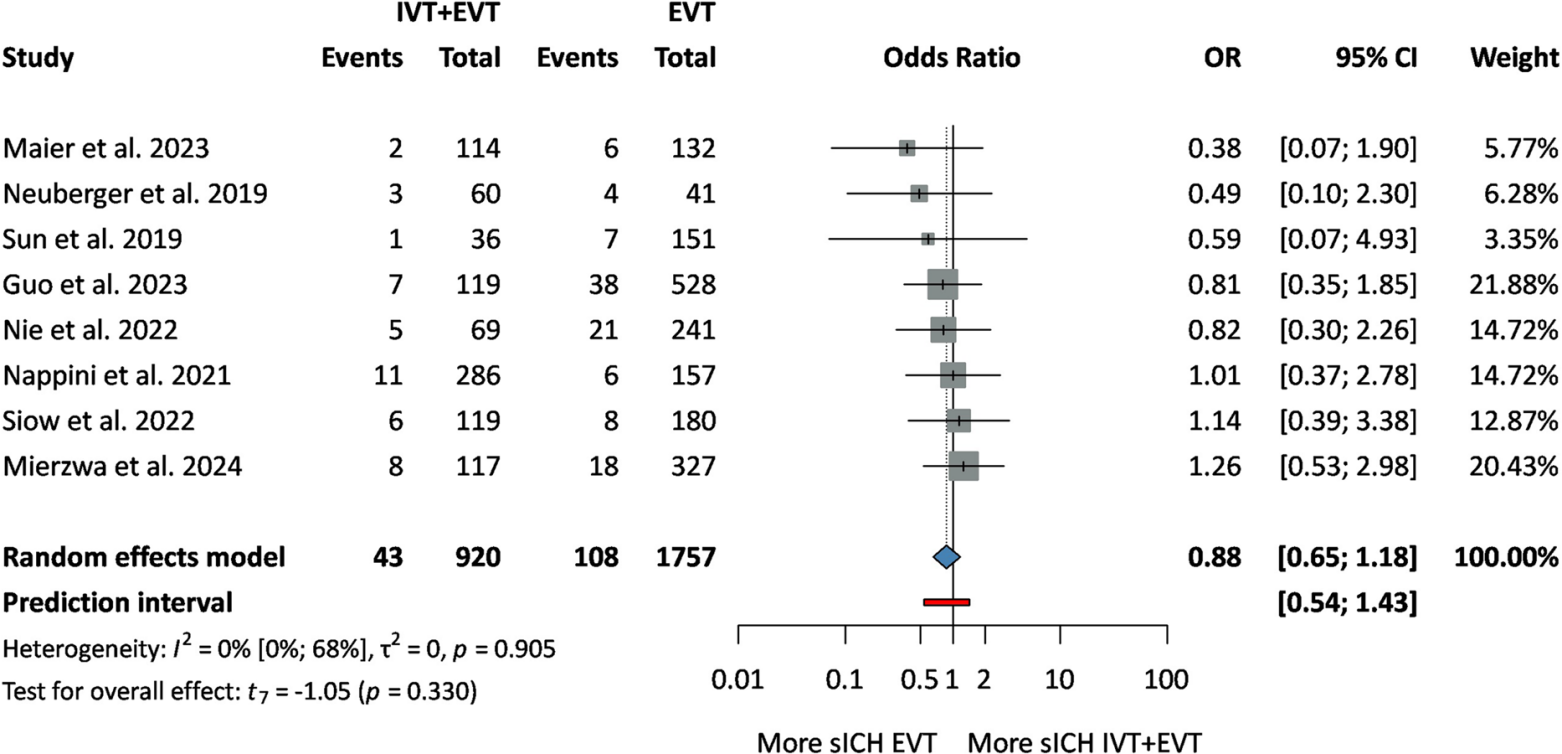
Forest plot representing the odds ratio (OR) of symptomatic intracranial hemorrhage

Eight studies reported sICH among 920 patients who underwent bridging therapy and 1,757 patients who underwent direct EVT. Across studies, sICH was defined as intracranial hemorrhage clinically manifested by neurological deterioration and imaging up to 48 h after treatment. Although patients who received bridging therapy had a lower occurrence of sICH, the association was not statistically significant (OR, 0.88; 95% CI, 0.65–1.18; *p* = 0.330). There was low heterogeneity among the included studies (*p* for heterogeneity = 0.905; *I*^2^ = 0%) (Fig. 4).

### Any type of intracranial hemorrhage

**Fig. 5:**
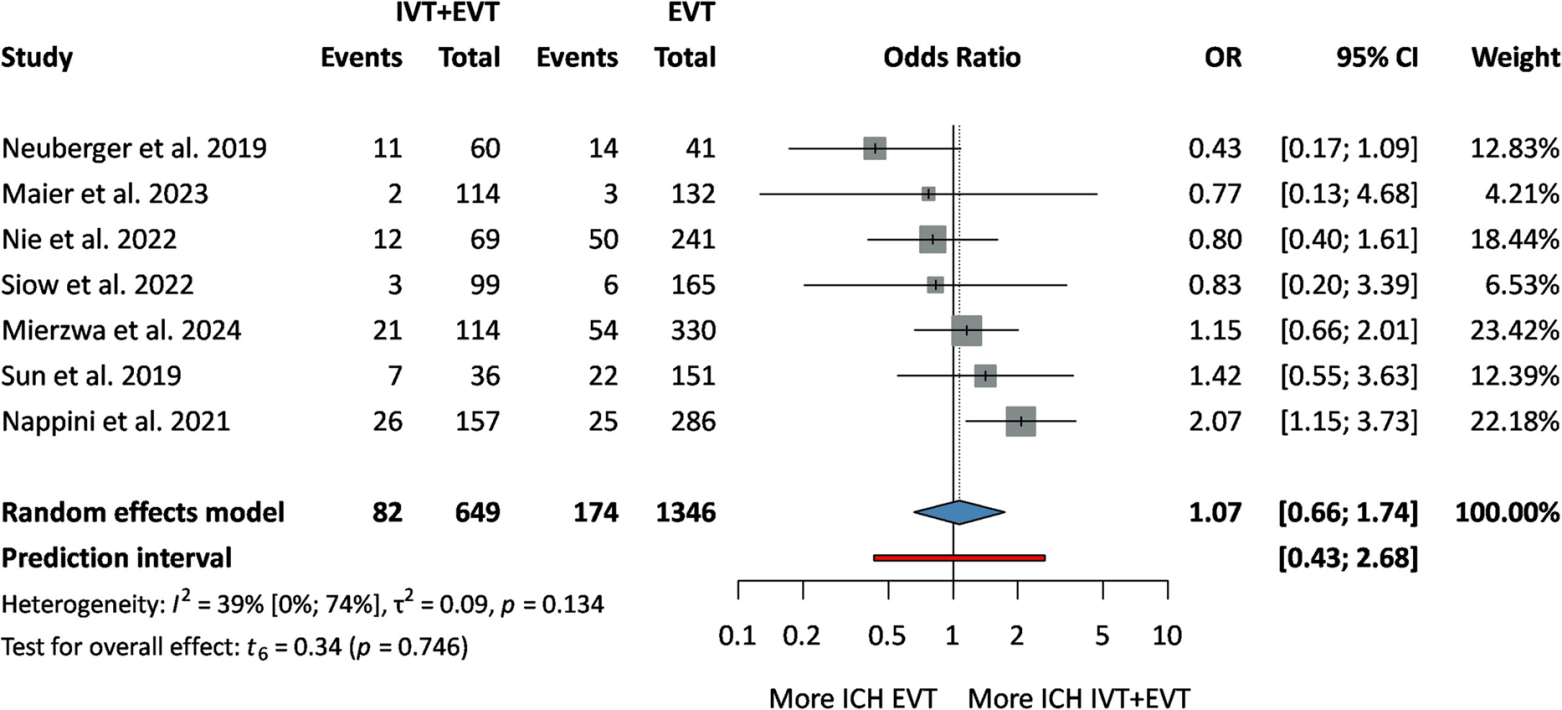
Forest plot representing the odds ratio (OR) of any intracranial hemorrhage

Seven studies reported any type of ICH among 649 patients who underwent bridging therapy and 1,346 patients who underwent direct EVT. ICH was defined across studies as hemorrhage manifested by imaging up to 48 h after treatment, and not necessarily a clinical manifestation of hemorrhage. There were no significant differences between patients who underwent bridging therapy and those who underwent direct EVT in terms of the occurrence of any type of ICH (OR, 1.07; 95% CI, 0.66–1.74; *p* = 0.746). There was moderate heterogeneity among the included studies (*p* for heterogeneity = 0.134; *I*^2^ = 39%) (Fig. 5).

### Mortality

**Fig. 6:**
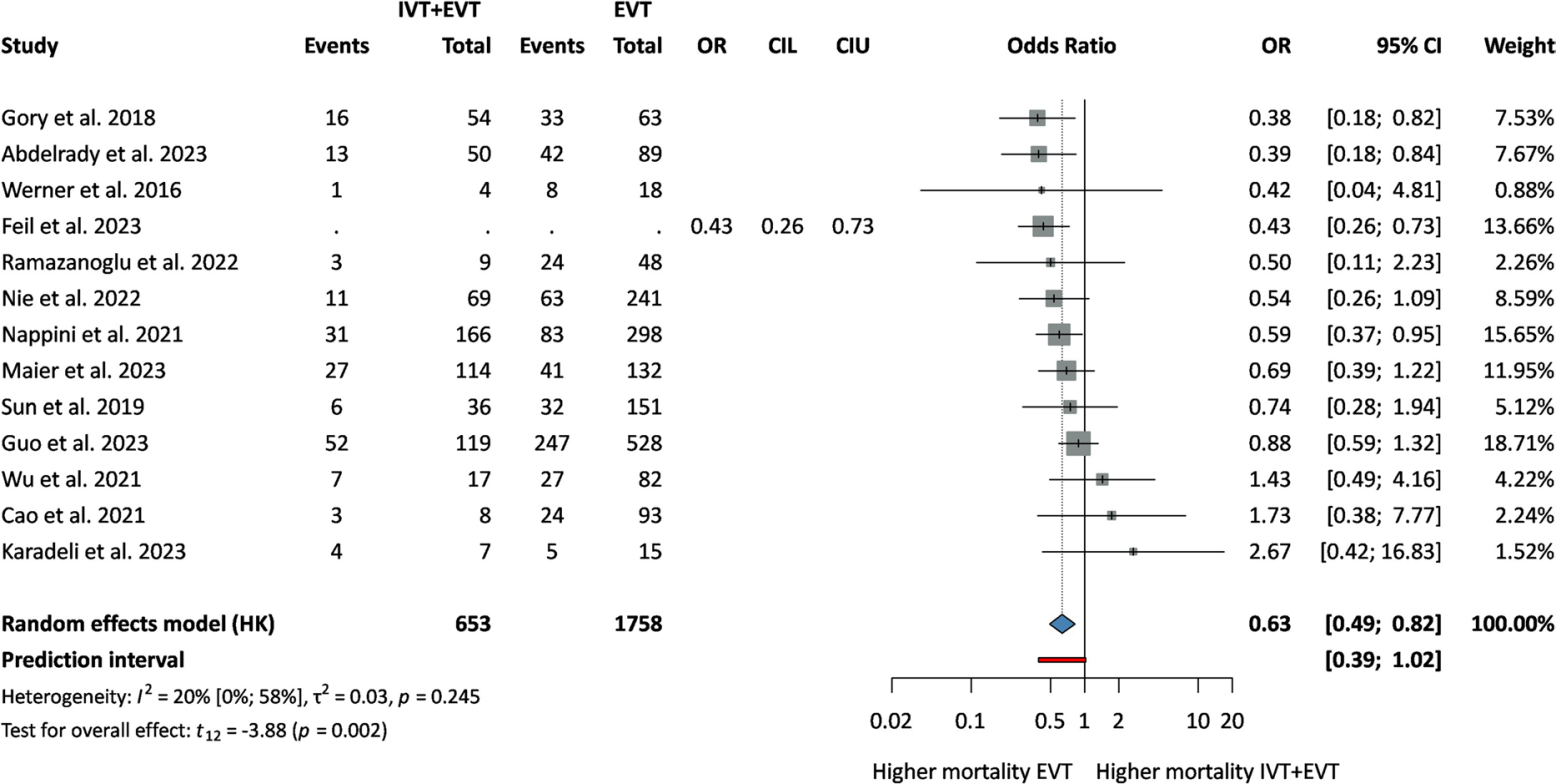
Forest plot representing the odds ratio (OR) of 90-day mortality

Thirteen studies reported 90-day mortality among 653 patients who underwent bridging therapy and 1758 patients who underwent direct EVT. Across studies, mortality was defined as death reported 90 days after treatment. Patients who underwent bridging therapy were less Likely to die at 90days (OR, 0.63; 95% CI, 0.49–0.82; *p* = 0.002). There was low heterogeneity among the included studies (*p* for heterogeneity = 0.245; *I*^2^ = 20%) (Fig. 6).

### Subgroup analysis

Subgroup analyses were conducted post hoc to explore potential effect modifiers, with differences assessed using p-values for subgroup differences. No adjustments for multiple comparisons were made, given the hypothesis-generating nature of these analyses.

The subgroup analysis showed various characteristics associated with the treatment effect. Patients with milder strokes (NIHSS 5–15) treated with bridging therapy had significantly higher odds of independent ambulation (OR, 1.52; 95% CI, 1.11–2.07; *p* for subgroup difference = 0.028), whereas the odds of functional independence (OR, 1.52; 95% CI, 1.17–1.66) were not significantly different across subgroups (*p* = 0.765) (sFigure 2–7). The OR decreased with increasing stroke severity for both functional independence and independent ambulation, though not significantly for functional independence.

Patients treated with bridging therapy within 24 h of symptom onset exhibited similar odds of functional independence (OR, 1.32; 95% CI, 1.11–1.58; *p*-value for test of subgroup differences = 0.709) and independent ambulation (OR, 1.25; 95% CI, 1.07–1.45; *p*-value for test of subgroup differences = 0.076) when compared to patients treated within 12 h of symptom onset (sFigure 8–13).

Treatment benefits of the bridging therapy compared to the direct EVT in terms of functional independence were shown in studies conducted both in Asia (OR, 1.39; 95% CI, 1.02–1.91) and in Europe and North America (OR, 1.66; 95% CI, 1.33–2.08; *p*-value for the test of subgroup differences = 0.308) (sFigure 14–19). Treatment benefits of the bridging therapy compared to the direct EVT were seen both among the studies that involved patients with BAO and those that involved patients with BAO and vertebral artery occlusion (sFigure 20–25).

**Fig. 7:**
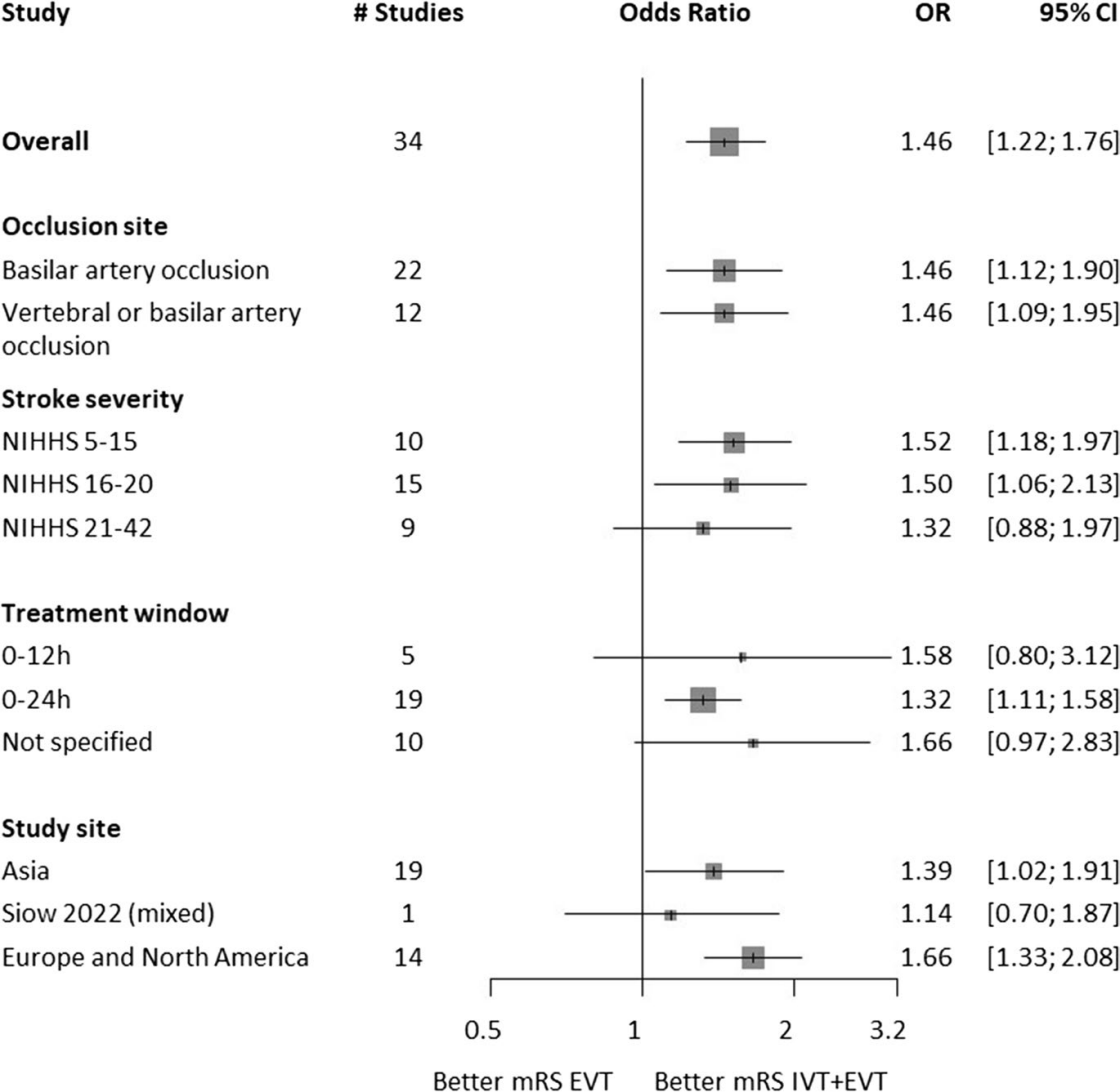
Summary plot of all subgroup analysis for the outcome of functional independence (90-day modified Rankin Scale 0–2)

As only four studies had poolable adjusted odds ratios, a limited analysis could be conducted, but no significant differences were found between the treatment groups (sFigure 26–29). A summary of all subgroup analyses for the outcome of functional independence is presented in Figure 7.

### Risk of bias assessment

Most studies included carried a moderate risk of bias. Of the fifty-eight studies involved in the meta-analysis, three studies (5.17%) had a high risk of bias, and fifty-five studies (94.83%) had a moderate risk of bias (sTable 1).

### Publication bias

The funnel plots were mostly symmetric, indicating a low risk of publication bias and suggesting a minimal likelihood of missing studies with negative or null results (sFigure 30).

### Quality of evidence

As we included only cohort studies, the certainty of evidence ranged from moderate to high for each outcome (sTable 2).

## Discussion

The aim of this meta-analysis was to compare two treatment strategies, bridging IVT and EVT versus direct EVT, in patients with BAO. Therefore, we analyzed the efficacy outcomes, such as 90-day functional independence, 90-day independent ambulation, and successful recanalization rate, as well as safety outcomes, such as symptomatic intracranial hemorrhage, any intracranial hemorrhage, and 90-day mortality. The results showed that patients who received bridging therapy were more Likely to achieve functional independence and had a lower mortality rate at 90days compared to those who received direct EVT. There were no significant differences between the two treatment strategies in terms of successful recanalization, symptomatic intracranial hemorrhage, and any type of hemorrhage.

Our findings are in line with those of previous meta-analyses in smaller cohorts, indicating that bridging IVT and EVT may result in better functional outcomes and lower mortality without increasing hemorrhagic complication rates [83, 84]. The European Stroke Organisation (ESO) has very recently updated treatment guidelines for BAO that recommend IVT before EVT for patients presenting within 4.5 h of symptom onset (class I, level A), and direct EVT for patients presenting from 4.5 to 24 h of symptom onset (class I, level A) [13]. However, decision-making on a case-by-case basis using advanced imaging techniques to identify viable brain tissue is recommended for patients beyond the treatment window for IVT (class IIa, level B) and EVT (class IIb, level C) [13].

Several randomized controlled trials and prospective registry studies have compared the treatment of BAO with EVT versus the best medical treatment (BMT) [6–9, 85]. The BASICS trial had a significantly higher percentage of patients receiving IVT before EVT than the BAOCHE, ATTENTION, and BEST trials [6]. This is mainly due to differences in treatment time windows: BASICS included patients within 6 h of estimated BAO onset, whereas BEST included patients within 8 h, ATTENTION included patients within 12 h, and BAOCHE included patients within 6 to 24 h of estimated BAO onset [6–9]. In addition, the BAOCHE, ATTENTION, and BEST trials involved patients from China, where IVT requires upfront payment [7–9]. In contrast, the BASICS trial was conducted in the Netherlands, where IVT costs are reimbursed [6]. The higher rates of IVT observed in the BASICS trial can be attributed to the reimbursement model, which facilitates timely treatment and is crucial to maximizing the benefits of thrombolysis [6].

Bridging IVT before EVT carries both potential risks and benefits [86]. The benefits include early reperfusion in microvascular areas that thrombectomy devices cannot reach and maintenance of downstream microvascular patency by reducing fibrinogen-dependent platelet aggregation with alteplase [86, 87]. In addition, IVT can facilitate clot removal and aspiration by separating it from the endovascular surface and dissolving distal perioperative clots, thus potentially reducing the need for stent retrieval [88]. However, arguments against bridging IVT before EVT include increased risks of hemorrhage, thrombus fragmentation, compromise of distal perfusion, delayed therapy onset, restricted use of antithrombotic therapy, and higher costs [89].

DIRECT-SAFE, a multicenter randomized controlled trial, compared direct EVT to standard bridging IVT before EVT in patients with large vessel occlusion in the intracranial internal carotid artery, middle cerebral artery (M1 or M2), or basilar artery presenting within 4.5 h of symptom onset [89]. The study concluded that the non-inferiority of direct EVT compared to bridging therapy was not demonstrated and that bridging therapy should be recommended as the standard treatment [89]. In addition, in trials such as SWIFT DIRECT, SKIP, and MR CLEAN-NO IV, direct EVT showed neither superiority nor non-inferiority to IVT before EVT in terms of functional independence at 90 days [90–92]. Only two trials from China, DEVT and DIRECT-MT, demonstrated that direct EVT was non-inferior to bridging therapy [93, 94].

Several publications, including the BASILAR registry study, indicate that the time window for BAO reperfusion may be more flexible than the one for the anterior circulation [8]. Treatment guidelines for BAO are evolving, with a potential expansion of the IVT treatment window to align more closely with the EVT window [95]. This shift is supported by observational data, but confirmation from larger randomized controlled studies is needed. The BRIDGE-TNK (NCT04733742) trial in China and the RESILIENT DIRECT-TNK (NCT05199194) trial in Brazil are investigating whether intravenous tenecteplase before endovascular treatment improves outcomes in patients with large vessel occlusions within 4.5 h of symptom onset. Extending the treatment window, the POST-ETERNAL (NCT05105633) trial investigates whether tenecteplase administered within 24 h before thrombectomy offers superior functional outcomes in acute BAO compared to current standards. In addition, the ATTENTION-IV early (NCT05827042) trial in China is evaluating endovascular thrombectomy alone versus bridging with intravenous thrombolysis within 4.5 h in patients with acute BAO. These studies will provide critical insights into optimizing treatment strategies across different time frames and patient subgroups.

### Strengths and limitations

Our analysis strictly followed a pre-registered protocol, ensuring transparency and minimizing bias in our investigation of treatment strategies for BAO. The primary strength of this analysis is that it incorporates data from multiple cohort studies. The inclusion of a patient population larger than ever before (more than 9372 patients across 58 cohorts) allows for a more generalized understanding of treatment effects. In addition, the analysis highlights significant outcomes, such as improved functional independence and lower mortality rates associated with bridging IVT and EVT, which are crucial for clinical decision-making in acute stroke management.

However, several Limitations should be taken into account. All included prospective studies were observational and not randomized, and thus the potential for clinician selection bias in IVT decisions may have influenced the observed associations. There was a substantial clinical heterogeneity among studies due to differences in treatment protocols, imaging techniques, and inclusion criteria, which undermines and Limits the generalizability of our findings. One Limitation of the study is the potential bias arising from the fact that all patients receiving bridging therapy were treated within 4.5 h of symptom onset, whereas those receiving direct EVT were treated up to 24 h. This discrepancy could partly account for the observed benefits in mortality and functional outcomes, as well as similar rates of symptomatic intracranial hemorrhage between the two treatment groups. However, our results showing a similar efficacy in favor of bridging treatment both within 12 and within 24 h do not suggest a significant treatment time-associated bias. Furthermore, Nappini et al. [48] found that patients treated with bridging therapy within 6 h had lower mortality rates than those receiving EVT alone within the same time frame. In addition, in cases without significant baseline ischemia (posterior circulation ASPECTS ≥ 8), Strbian et al. [96] observed no significant association between onset-to-treatment time and poor outcomes. These findings suggest that although time is a critical factor, its impact may be nuanced by patient-specific factors and baseline ischemic burden, supporting the consideration of bridging therapy even within extended treatment windows.

### Implications for research

Future research on bridging IVT and EVT for BAO should incorporate several key design improvements to address existing limitations. Implementing stratified randomization based on critical factors such as stroke severity, time from symptom onset to treatment, and collateral circulation status is essential to reduce selection bias and achieve balanced treatment groups. In addition, standardized and long-term follow-up (12–24 months) with comprehensive neurological assessment is vital for capturing long-term outcomes of bridging therapy. At the same time, advanced imaging can minimize inter-observer variability in evaluating imaging-based criteria and outcomes. To further mitigate confounding, researchers should conduct multivariable analyses and sensitivity evaluations to account for critical variables such as pre-stroke disability, comorbidities, and variation in provided care. By incorporating these methodological improvements, future studies can generate more reliable evidence that informs optimized therapeutic strategies for BAO.

### Implications for clinical practice

Our findings suggest that identifying optimal patient subgroups who would benefit most from bridging therapy is crucial. Consideration should be given to factors such as patient characteristics, stroke severity, time to treatment, and collateral circulation. In addition, long-term follow-up is crucial to assessing the treatment effects and potential late complications.

## Conclusion

In conclusion, our study demonstrated that bridging IVT with EVT for patients with BAO leads to improved outcomes in terms of functional independence and lower mortality at 90 days compared to direct EVT without increasing the rate of symptomatic intracranial hemorrhage. The bridging treatment approach was shown to be beneficial across all subgroups. Our observational findings align with current guideline-based practice but should be interpreted with caution and considered hypothesis-generating rather than directive for clinical decision-making.

## Supplementary Information

Below is the link to the electronic supplementary material.

ESM 1 (DOCX 877 KB)

ESM 2 (PDF 68.7 KB)

## Data Availability

The data that support the findings of this study are available from the corresponding author upon reasonable request.
