# Supplementary material for: Bridging therapy versus direct endovascular thrombectomy in basilar artery occlusion stroke: a systematic review and meta-analysis
Source: GeroScience. 2025 Sep 18;48(3):4099–120. doi: 10.1007/s11357-025-01887-0 (PMC13356182; doi:10.1007/s11357-025-01887-0)
Supplement: Supplementary file 1 — (DOCX 877 KB) [file 11357_2025_1887_MOESM1_ESM.docx]

**SUPPLEMENTARY ONLINE MATERIAL**

**Bridging therapy versus direct endovascular thrombectomy in basilar artery stroke:**

**a systematic review and meta-analysis**

**Authors:**

Esra Zhubi, Azamat Bissenov, Marie Anne Engh, Réka Tóth, András Attila Horváth, Peter Hegyi, Bence Gunda*

***Corresponding author:**

Bence Gunda MD, PhD

Postal address: H-1083 Budapest, Balassa utca 6, Hungary

Tel.: +36-1-2100330

E-mail address: bence.gunda@gmail.com

**SUMMARY**

1. **Supplementary Figures**

**Supplementary Figure 1.** PRISMA Flowchart of study search and selection.

**Supplementary Figure 2**. Forest plot representing the odds ratio (OR) of functional independence (90-day modified Rankin Scale 0-2) based on stroke severity.

**Supplementary Figure 3.** Forest plot representing the odds ratio (OR) of independent ambulation (90-day modified Rankin Scale 0-3) based on stroke severity.

**Supplementary Figure 4.** Forest plot representing the odds ratio (OR) of successful recanalization based on stroke severity.

**Supplementary Figure 5.** Forest plot representing the odds ratio (OR) of symptomatic intracranial hemorrhage based on stroke severity.

**Supplementary Figure 6.** Forest plot representing the odds ratio (OR) of any intracranial hemorrhage based on stroke severity.

**Supplementary Figure 7.** Forest plot representing the odds ratio (OR) of 90-day mortality based on stroke severity based on stroke severity.

**Supplementary Figure 8**. Forest plot representing the odds ratio (OR) of functional independence (90-day modified Rankin Scale 0-2) based on the treatment window.

**Supplementary Figure 9.** Forest plot representing the odds ratio (OR) of independent ambulation (90-day modified Rankin Scale 0-3) based on the treatment window.

**Supplementary Figure 10.** Forest plot representing the odds ratio (OR) of successful recanalization based on the treatment window.

**Supplementary Figure 11.** Forest plot representing the odds ratio (OR) of symptomatic intracranial hemorrhage based on the treatment window.

**Supplementary Figure 12.** Forest plot representing the odds ratio (OR) of any intracranial hemorrhage based on the treatment window.

**Supplementary Figure 13.** Forest plot representing the odds ratio (OR) of 90-day mortality based on the treatment window.

**Supplementary Figure 14**. Forest plot representing the odds ratio (OR) of functional independence (90-day modified Rankin Scale 0-2) based on the study site.

**Supplementary Figure 15.** Forest plot representing the odds ratio (OR) of independent ambulation (90-day modified Rankin Scale 0-3) based on the study site.

**Supplementary Figure 16.** Forest plot representing the odds ratio (OR) of successful recanalization based on the study site.

**Supplementary Figure 17.** Forest plot representing the odds ratio (OR) of symptomatic intracranial hemorrhage based on the study site.

**Supplementary Figure 18.** Forest plot representing the odds ratio (OR) of any intracranial hemorrhage based on the study site.

**Supplementary Figure 19.** Forest plot representing the odds ratio (OR) of 90-day mortality based on the study site.

**Supplementary Figure 20.** Forest plot representing the odds ratio (OR) of functional independence (90-day modified Rankin Scale 0-2) based on the occlusion site.

**Supplementary Figure 21.** Forest plot representing the odds ratio (OR) of independent ambulation (90-day modified Rankin Scale 0-3) based on the occlusion site.

**Supplementary Figure 22.** Forest plot representing the odds ratio (OR) of successful recanalization based on the occlusion site.

**Supplementary Figure 23.** Forest plot representing the odds ratio (OR) of symptomatic intracranial hemorrhage based on the occlusion site.

**Supplementary Figure 24.** Forest plot representing the odds ratio (OR) of any intracranial hemorrhage based on the occlusion site.

**Supplementary Figure 25.** Forest plot representing the odds ratio (OR) of 90-day mortality based on the occlusion site.

**Supplementary Figure 26**. Forest plot representing the adjusted odds ratio (OR) of functional independence (90-day modified Rankin Scale 0-2) based on stroke severity.

**Supplementary Figure 27**. Forest plot representing the adjusted odds ratio (OR) of functional independence (90-day modified Rankin Scale 0-2) based on the treatment window.

**Supplementary Figure 28.** Forest plot representing the adjusted odds ratio (OR) of functional independence (90-day modified Rankin Scale 0-2) based on the study site.

**Supplementary Figure 29.** Forest plot representing the adjusted odds ratio (OR) of functional independence (90-day modified Rankin Scale 0-2) based on the occlusion site.

**Supplementary Figure 30**. Funnel plot for publication bias: a) 90-day functional independence b) 90-day independent ambulation c) successful recanalization rate d) symptomatic intracranial hemorrhage e) any intracranial hemorrhage f) 90-day mortality.

1. **Supplementary Tables**

**Supplementary Table 1.** Risk of bias assessment using ROBINS-1 tool. (Risk Of Bias in Non-randomized Studies - of Interventions)

**Supplementary Table 2**. Grading of Recommendations, Assessment, Development and Evaluations (GRADE) assessment.

1. **Studies involved in the meta-analysis (References)**
2. **Supplementary Figures**
   1. **Supplementary Figure 1.** PRISMA Flowchart of study search and selection.

**
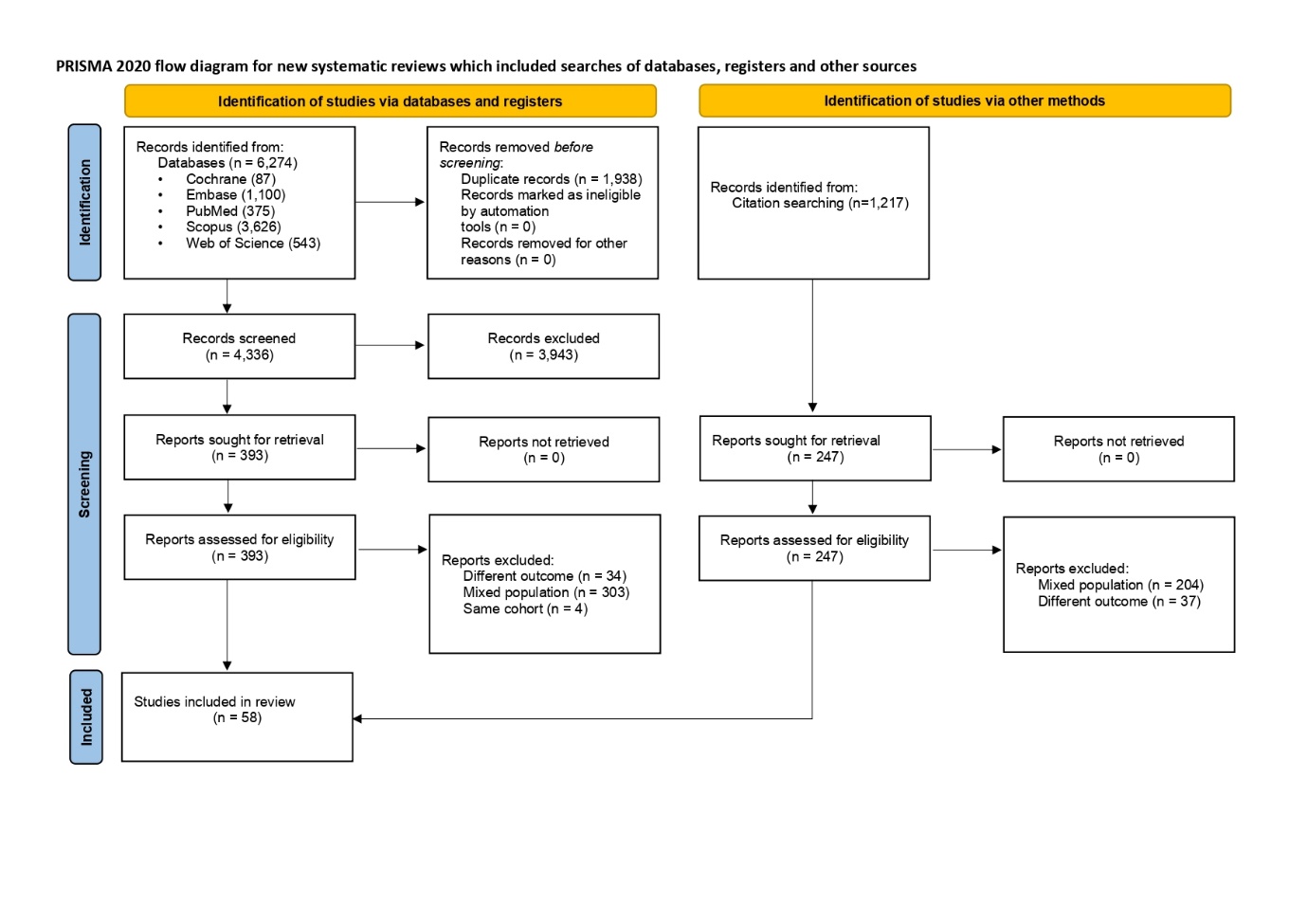
**

- 1. **Supplementary Figure 2**. Forest plot representing the odds ratio (OR) of functional independence (90-day modified Rankin Scale 0-2) based on stroke severity.

- 1. **Supplementary Figure 3.** Forest plot representing the odds ratio (OR) of independent ambulation (90-day modified Rankin Scale 0-3) based on stroke severity.

- 1. **Supplementary Figure 4.** Forest plot representing the odds ratio (OR) of successful recanalization based on stroke severity.

- 1. **Supplementary Figure 5.** Forest plot representing the odds ratio (OR) of symptomatic intracranial hemorrhage based on stroke severity.

- 1. **Supplementary Figure 6.** Forest plot representing the odds ratio (OR) of any intracranial hemorrhage based on stroke severity.

- 1. **Supplementary Figure 7.** Forest plot representing the odds ratio (OR) of 90-day mortality based on stroke severity based on stroke severity.

- 1. **Supplementary Figure 8**. Forest plot representing the odds ratio (OR) of functional independence (90-day modified Rankin Scale 0-2) based on the treatment window.

- 1. **Supplementary Figure 9.** Forest plot representing the odds ratio (OR) of independent ambulation (90-day modified Rankin Scale 0-3) based on the treatment window.

- 1. **Supplementary Figure 10.** Forest plot representing the odds ratio (OR) of successful recanalization based on the treatment window.

- 1. **Supplementary Figure 11.** Forest plot representing the odds ratio (OR) of symptomatic intracranial hemorrhage based on the treatment window.

- 1. **Supplementary Figure 12.** Forest plot representing the odds ratio (OR) of any intracranial hemorrhage based on the treatment window.

- 1. **Supplementary Figure 13.** Forest plot representing the odds ratio (OR) of 90-day mortality based on the treatment window.

- 1. **Supplementary Figure 14**. Forest plot representing the odds ratio (OR) of functional independence (90-day modified Rankin Scale 0-2) based on the study site.

- 1. **Supplementary Figure 15.** Forest plot representing the odds ratio (OR) of independent ambulation (90-day modified Rankin Scale 0-3) based on the study site.

- 1. **Supplementary Figure 16.** Forest plot representing the odds ratio (OR) of successful recanalization based on the study site.

- 1. **Supplementary Figure 17.** Forest plot representing the odds ratio (OR) of symptomatic intracranial hemorrhage based on the study site.

- 1. **Supplementary Figure 18.** Forest plot representing the odds ratio (OR) of any intracranial hemorrhage based on the study site.

- 1. **Supplementary Figure 19.** Forest plot representing the odds ratio (OR) of 90-day mortality based on the study site.

- 1. **Supplementary Figure 20.** Forest plot representing the adjusted odds ratio (OR) of functional independence (90-day modified Rankin Scale 0-2) based on the occlusion site.

- 1. **Supplementary Figure 21.** Forest plot representing the adjusted odds ratio (OR) of independent ambulation (90-day modified Rankin Scale 0-3) based on the occlusion site.

- 1. **Supplementary Figure 22.** Forest plot representing the adjusted odds ratio (OR) of successful recanalization based on the occlusion site.

- 1. **Supplementary Figure 23.** Forest plot representing the adjusted odds ratio (OR) of symptomatic intracranial hemorrhage based on the occlusion site.

- 1. **Supplementary Figure 24.** Forest plot representing the adjusted odds ratio (OR) of any intracranial hemorrhage based on the occlusion site.

- 1. **Supplementary Figure 25.** Forest plot representing the adjusted odds ratio (OR) of 90-day mortality based on the occlusion site.

- 1. **Supplementary Figure 26**. Forest plot representing the adjusted odds ratio (OR) of functional independence (90-day modified Rankin Scale 0-2) based on stroke severity.


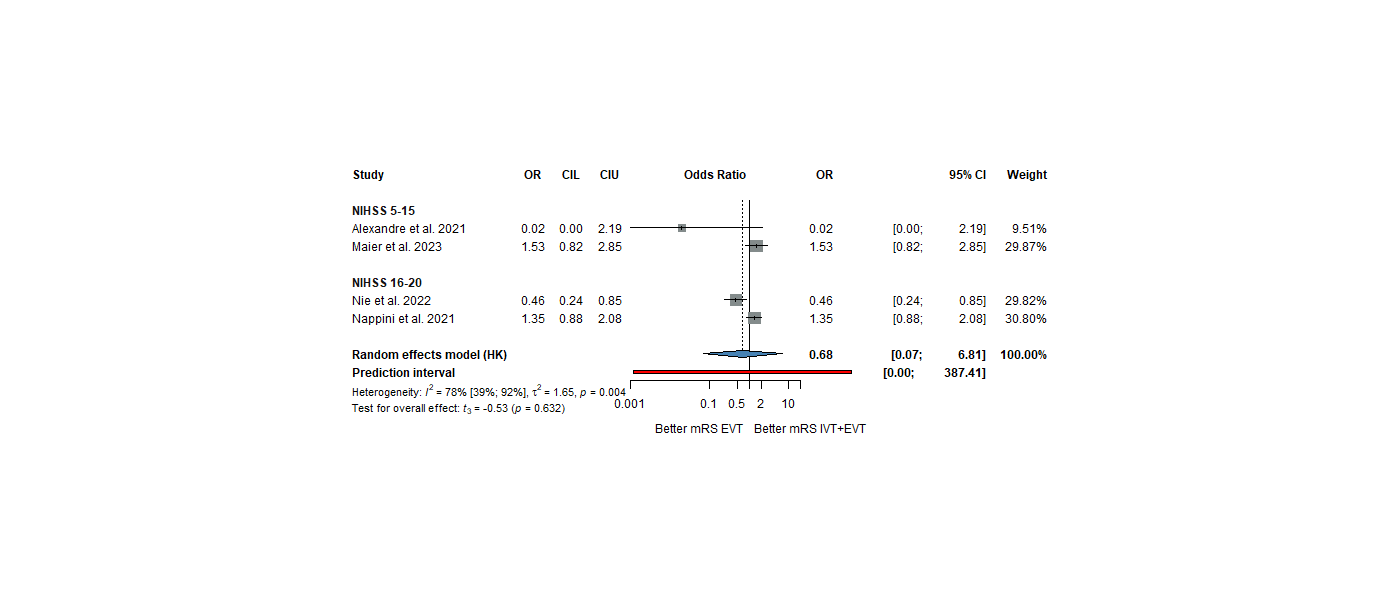


- 1. **Supplementary Figure 27**. Forest plot representing the adjusted odds ratio (OR) of functional independence (90-day modified Rankin Scale 0-2) based on the treatment window.


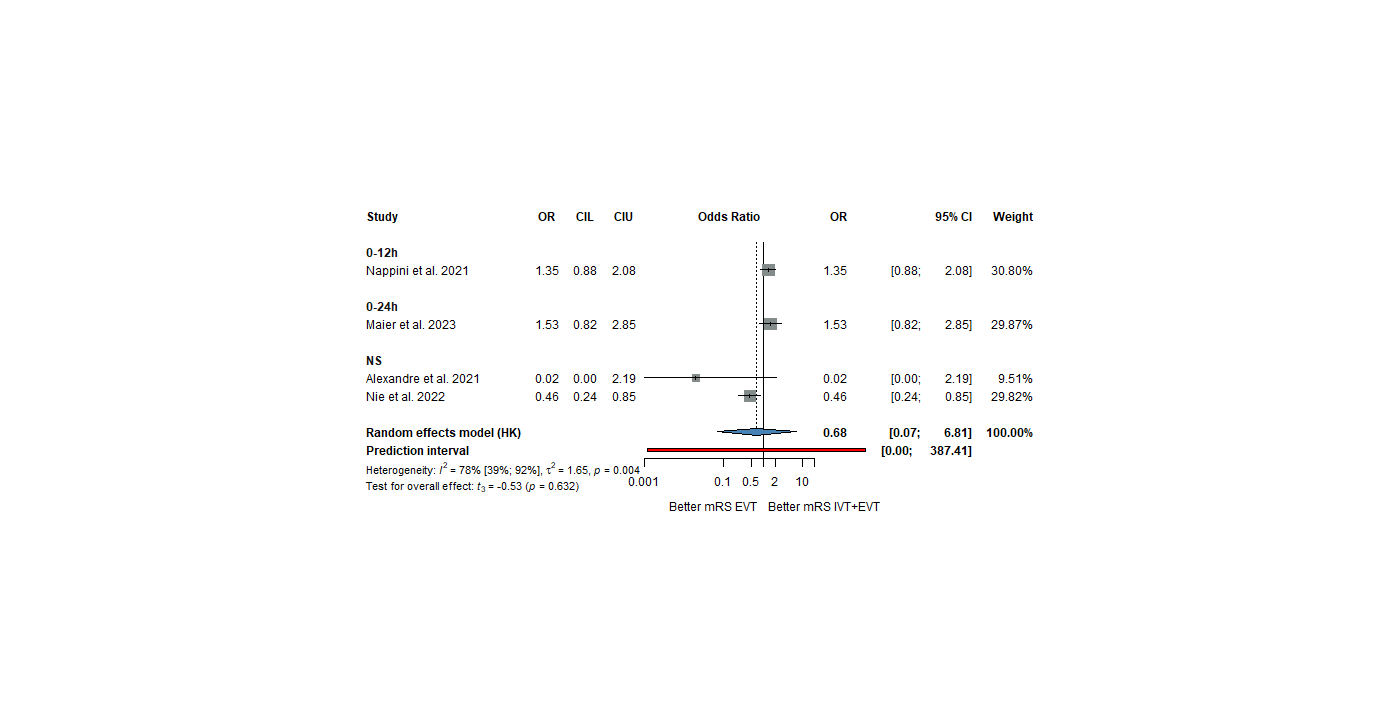


- 1. **Supplementary Figure 28.** Forest plot representing the adjusted odds ratio (OR) of functional independence (90-day modified Rankin Scale 0-2) based on the study site.

**
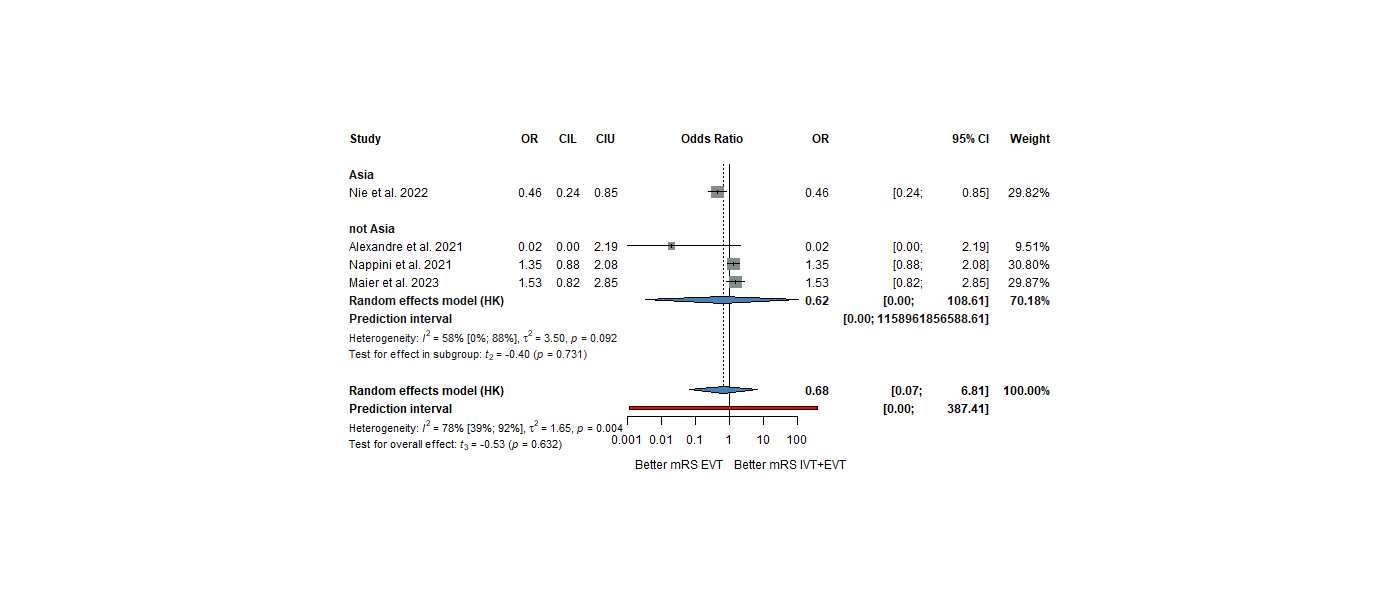
**

- 1. **Supplementary Figure 29.** Forest plot representing the adjusted odds ratio (OR) of functional independence (90-day modified Rankin Scale 0-2) based on the occlusion site.


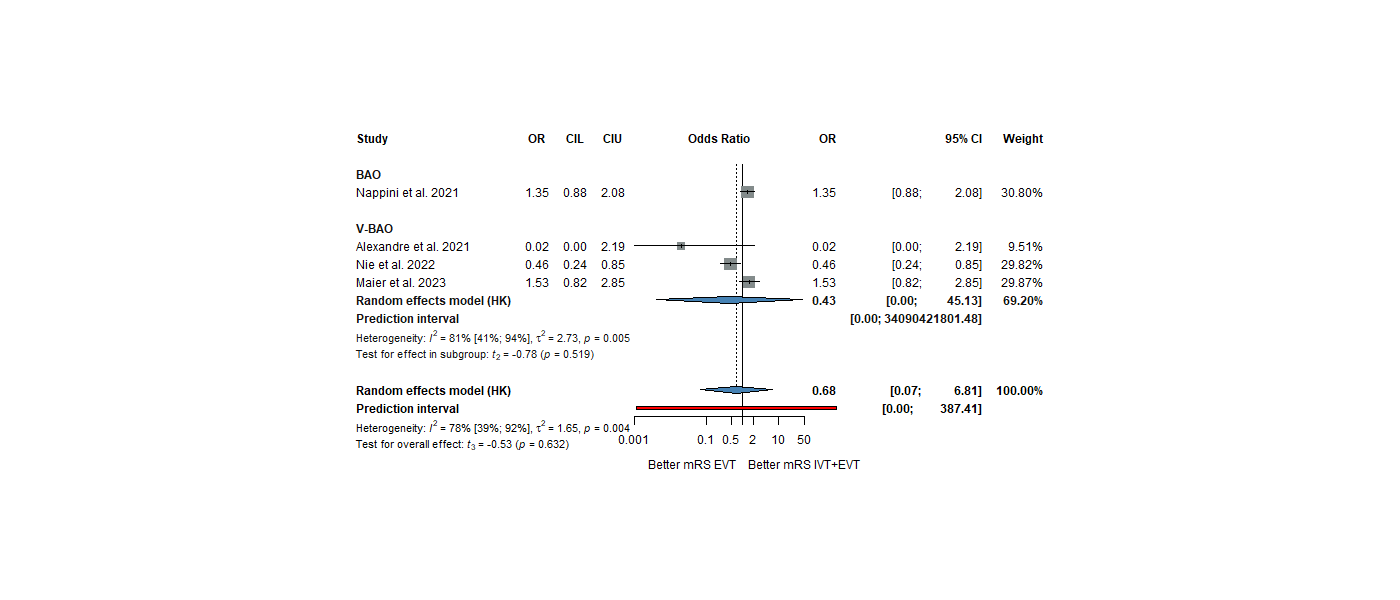


- 1. **Supplementary Figure 30**. Funnel plot for publication bias: a) 90-day functional independence b) 90-day independent ambulation c) successful recanalization rate d) symptomatic intracranial hemorrhage e) any intracranial hemorrhage f) 90-day mortality.

a)


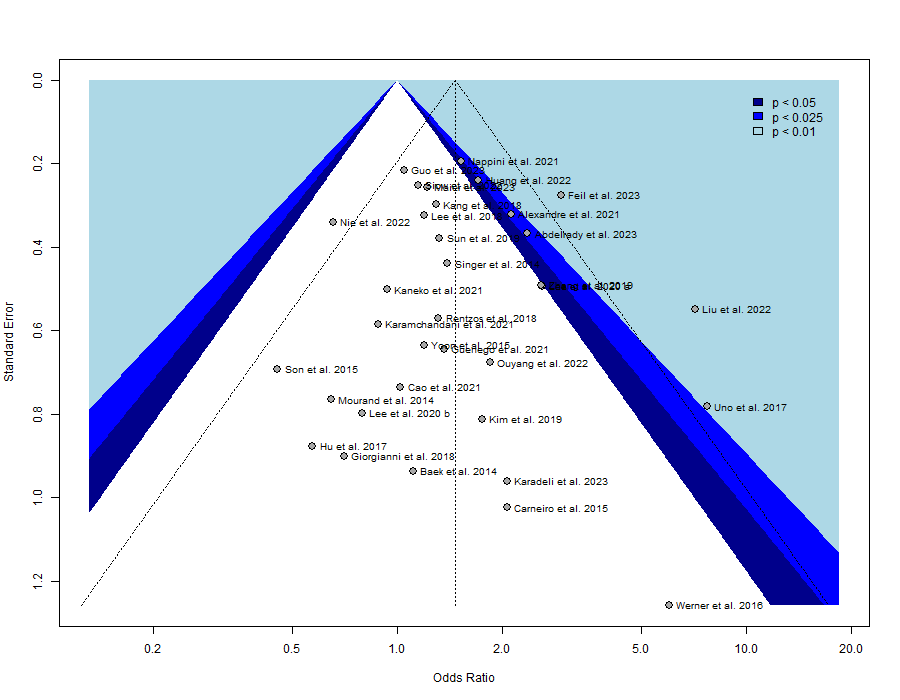


b)


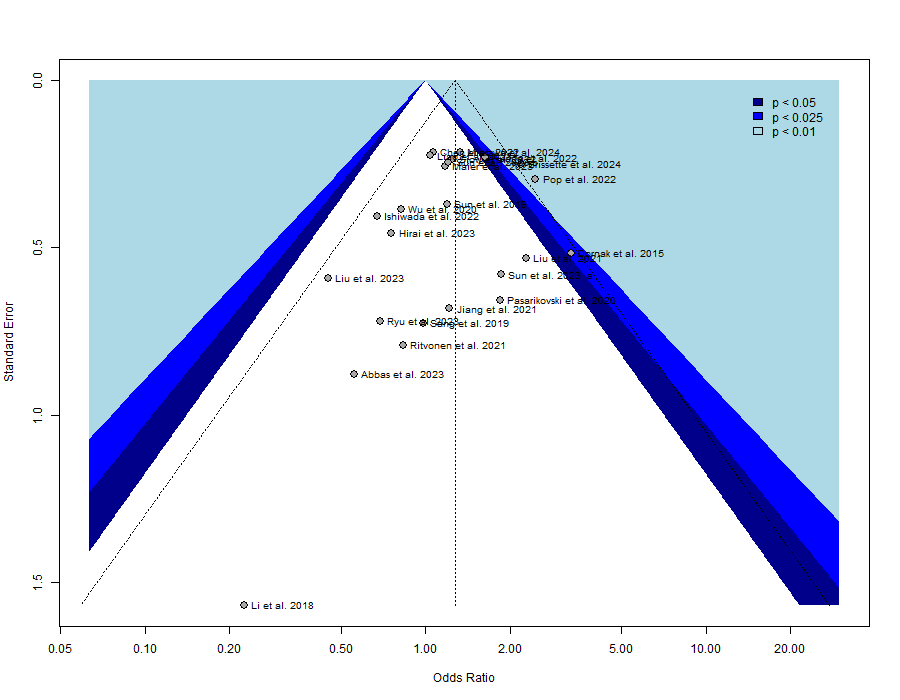


c)


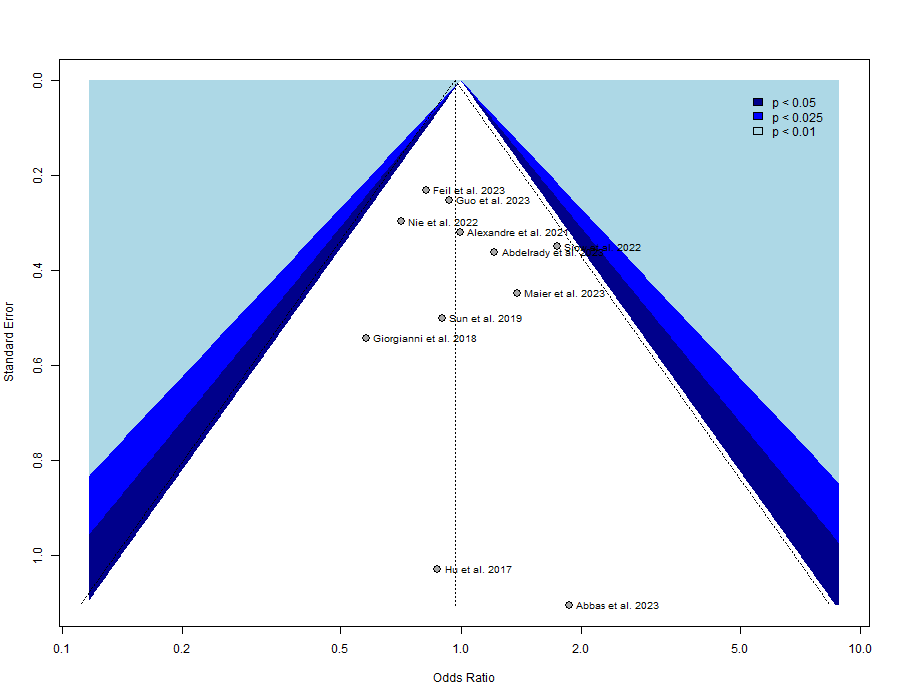


d)


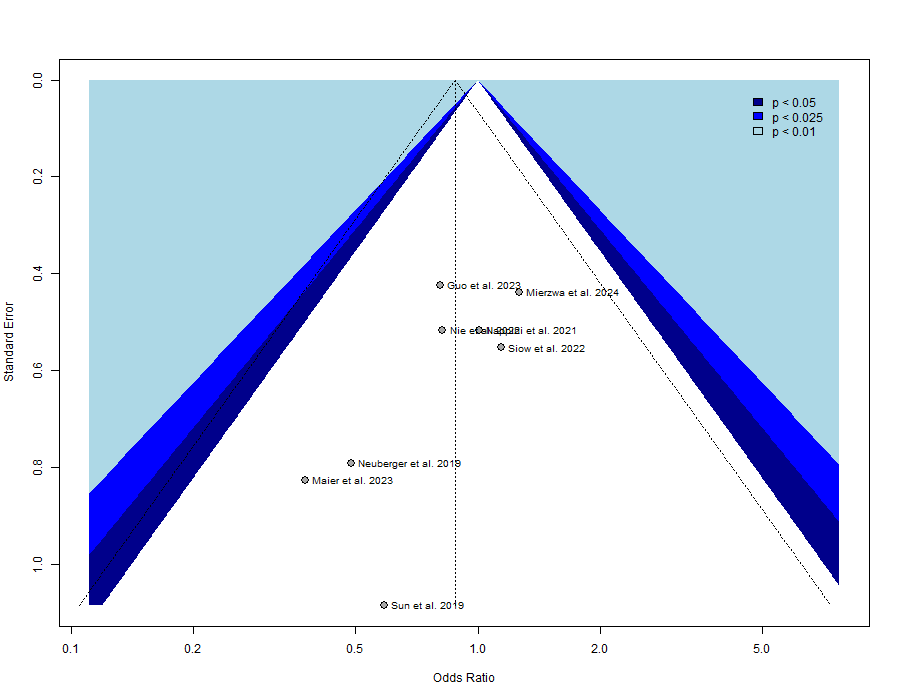


e)


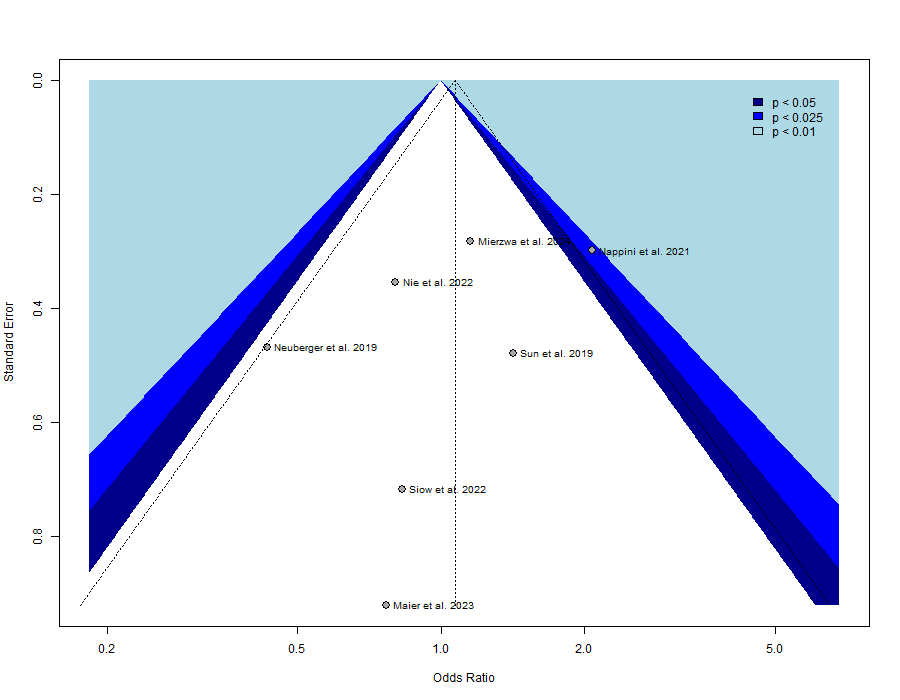


f)

**
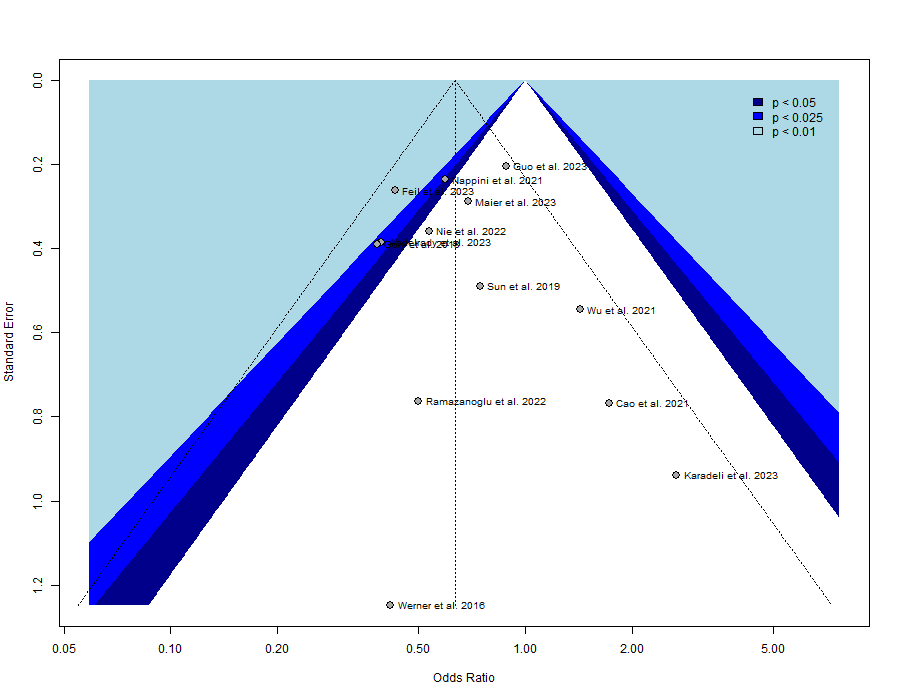
**

1. **Supplementary Tables**

**Supplementary Table 1.** Risk of bias assessment using ROBINS-1 tool. (Risk Of Bias in Non-randomized Studies - of Interventions)


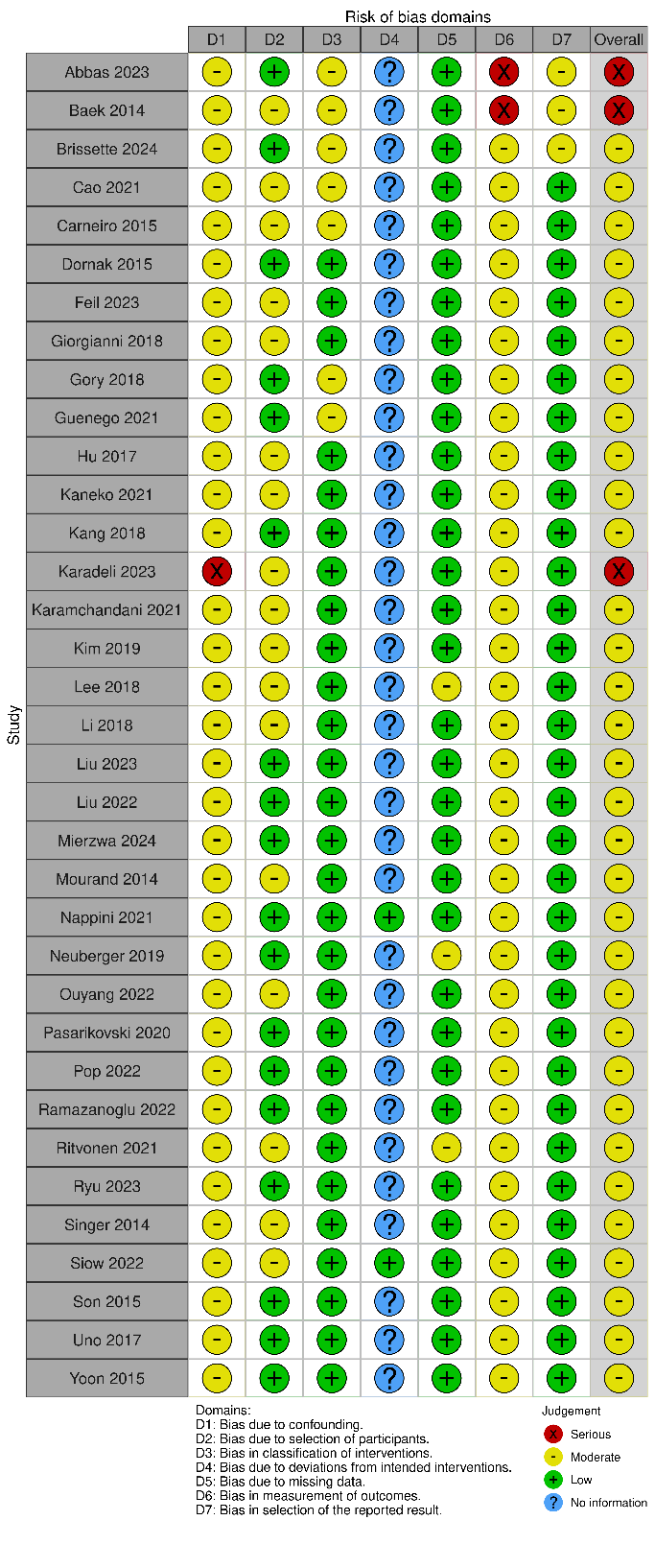

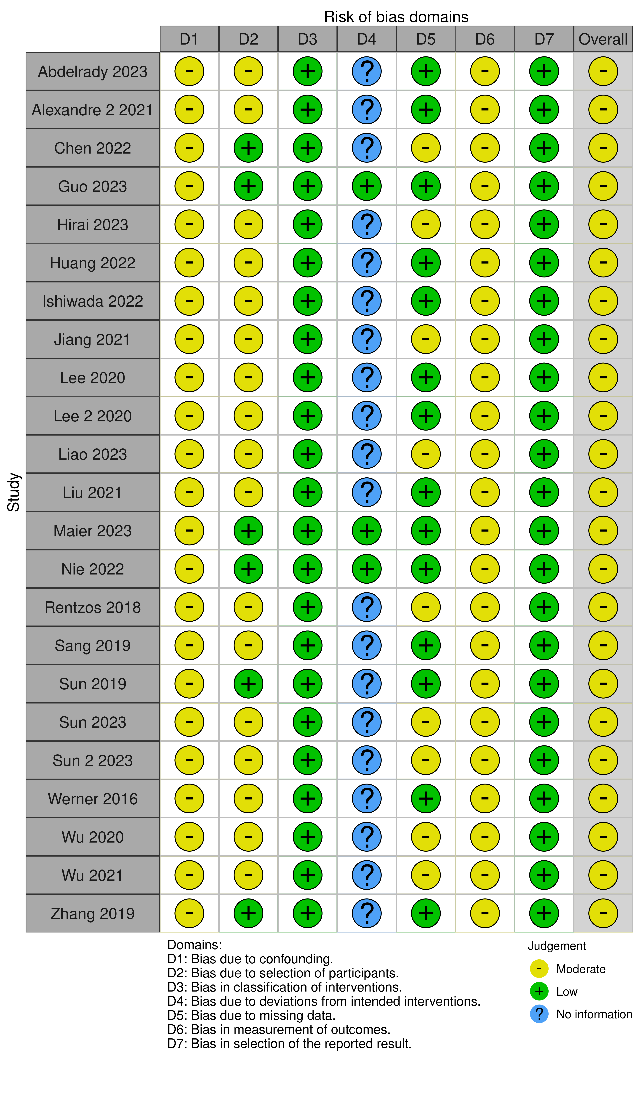


**Supplementary Table 2**. GRADE assessment. (GRADE: Grading of Recommendations Assessment, Development and Evaluation)

| **Certainty assessment** | | | | | | | **№ of patients** | | **Effect** | | **Certainty** | **Importance** |
| --- | --- | --- | --- | --- | --- | --- | --- | --- | --- | --- | --- | --- |
| **№ of studies** | **Study design** | **Risk of bias** | **Inconsistency** | **Indirectness** | **Imprecision** | **Other considerations** | **bridging therapy** | **endovascular treatment alone** | **Relative (95% CI)** | **Absolute (95% CI)** |  |  |
| **90-day good functional outcome (assessed with: mRS: 0-2)** | | | | | | | | | | | | |
| 34 | non-randomised studies | not serious | not serious | not serious | not serious | none | 567/1355 (41.8%) | 1134/3336 (34.0%) | **OR 1.46** (1.22 to 1.76) | **89 more per 1,000** (from 46 more to 136 more) | ⨁⨁⨁⨁ High | CRITICAL |
| **90-day good functional outcome (assessed with: mRS: 0-3)** | | | | | | | | | | | | |
| 24 | non-randomised studies | not serious | not serious | not serious | not serious | none | 577/1244 (46.4%) | 1385/3500 (39.6%) | **OR 1.27** (1.07 to 1.52) | **58 more per 1,000** (from 16 more to 103 more) | ⨁⨁⨁⨁ High | CRITICAL |
| **Successful recanalization rate (assessed with: mTICI: 2b-3)** | | | | | | | | | | | | |
| 11 | non-randomised studies | not serious | not serious | not serious | serious^a^ | none | 719/905 (79.4%) | 1525/1938 (78.7%) | **OR 0.97** (0.79 to 1.18) | **5 fewer per 1,000** (from 42 fewer to 26 more) | ⨁⨁⨁◯ Moderate | CRITICAL |
| **Symptomatic intracranial hemorrhage (assessed with: yes/no)** | | | | | | | | | | | | |
| 8 | non-randomised studies | not serious | not serious | not serious | serious^a^ | none | 43/920 (4.7%) | 108/1757 (6.1%) | **OR 0.88** (0.65 to 1.18) | **7 fewer per 1,000** (from 21 fewer to 10 more) | ⨁⨁⨁◯ Moderate | CRITICAL |
| **Any type of intracranial hemorrhage (assessed with: yes/no)** | | | | | | | | | | | | |
| 7 | non-randomised studies | not serious | not serious | not serious | serious^a^ | none | 82/649 (12.6%) | 174/1346 (12.9%) | **OR 1.07** (0.66 to 1.74) | **8 more per 1,000** (from 40 fewer to 76 more) | ⨁⨁⨁◯ Moderate | CRITICAL |
| **90-day mortality (assessed with: yes/no)** | | | | | | | | | | | | |
| 13 | non-randomised studies | not serious | not serious | not serious | not serious | none | 174/653 (26.6%) | 629/1758 (35.8%) | **OR 0.63** (0.49 to 0.82) | **98 fewer per 1,000** (from 143 fewer to 44 fewer) | ⨁⨁⨁⨁ High | CRITICAL |

**CI:** confidence interval; **OR:** odds ratio

1. **Studies Involved in the meta-analysis (References)**
2. Abbas, R., et al., *Mechanical Thrombectomy for Stroke Due to Acute Basilar Artery Occlusion, a Safety and Efficacy Analysis.* Neurosurgery, 2023. **92**(4): p. 772-778.
3. Baek, J.M., et al., *Acute basilar artery occlusion: outcome of mechanical thrombectomy with Solitaire stent within 8 hours of stroke onset.* AJNR Am J Neuroradiol, 2014. **35**(5): p. 989-93.
4. Brissette, V., et al., *Benefits of First Pass Recanalization in Basilar Strokes Based on Initial Clinical Severity.* Clin Neuroradiol, 2024.
5. Cao, J., et al., *Predictors of Functional Outcome and Mortality in Endovascular Treatment for Acute Basilar Artery Occlusion: A Single-Centre Experience.* Front Neurol, 2021. **12**: p. 731300.
6. Carneiro, A.A., et al., *Mechanical thrombectomy in patients with acute basilar occlusion using stent retrievers.* Interv Neuroradiol, 2015. **21**(6): p. 710-4.
7. Dorňák, T., et al., *Endovascular treatment of acute basilar artery occlusion: time to treatment is crucial.* Clin Radiol, 2015. **70**(5): p. e20-7.
8. Feil, K., et al., *Endovascular thrombectomy for basilar artery occlusion stroke: Analysis of the German Stroke Registry-Endovascular Treatment.* Eur J Neurol, 2023. **30**(5): p. 1293-1302.
9. Giorgianni, A., et al., *Endovascular Treatment of Acute Basilar Artery Occlusion: Registro Endovascolare Lombardo Occlusione Basilar Artery (RELOBA) Study Group Experience.* J Stroke Cerebrovasc Dis, 2018. **27**(9): p. 2367-2374.
10. Gory, B., et al., *Predictors for Mortality after Mechanical Thrombectomy of Acute Basilar Artery Occlusion.* Cerebrovasc Dis, 2018. **45**(1-2): p. 61-67.
11. Guenego, A., et al., *Thrombectomy for Basilar Artery Occlusion with Mild Symptoms.* World Neurosurg, 2021. **149**: p. e400-e414.
12. Hu, S.Y., et al., *Effectiveness and Safety of Mechanical Thrombectomy with Stent Retrievers in Basilar Artery Occlusion: Comparison with Anterior Circulation Occlusions.* J Korean Neurosurg Soc, 2017. **60**(6): p. 635-643.
13. Kaneko, J., et al., *Endovascular treatment of acute basilar artery occlusion: Outcomes, influencing factors and imaging characteristics from the Tama-REgistry of acute thrombectomy (TREAT) study.* J Clin Neurosci, 2021. **86**: p. 184-189.
14. Kang, D.H., et al., *Endovascular Thrombectomy for Acute Basilar Artery Occlusion: A Multicenter Retrospective Observational Study.* J Am Heart Assoc, 2018. **7**(14).
15. HH, K., *Factors Affecting Functional Outcomes and Mortality After Thrombectomy for Basilar Artery Occlusions: Recanalization Time and Collateral Scoring.* Turk J Neurol 2023. **29**: p. 12-17.
16. Karamchandani, R.R., et al., *Age and discharge modified Rankin score are associated with 90-Day functional outcome after basilar artery occlusion treated with endovascular therapy.* Interv Neuroradiol, 2021. **27**(4): p. 531-538.
17. Kim, J.G., et al., *DWI-pc-ASPECT score in basilar artery occlusion: is 6 points or less always indicative of a bad outcome?* Interv Neuroradiol, 2019. **25**(4): p. 371-379.
18. Lee, W.J., et al., *Impact of stroke mechanism in acute basilar occlusion with reperfusion therapy.* Ann Clin Transl Neurol, 2018. **5**(3): p. 357-368.
19. Li, C., et al., *Outcome of endovascular treatment for acute basilar artery occlusion in the modern era: a single institution experience.* Neuroradiology, 2018. **60**(6): p. 651-659.
20. Liu, X.L., et al., *Tmax profile in computed tomography perfusion-based RAPID software maps influences outcome after mechanical thrombectomy in patients with basilar artery occlusion.* J Neurointerv Surg, 2023. **15**(7): p. 639-643.
21. Liu, L., et al., *Prognostic value of pretreatment diffusion-weighted imaging score for acute basilar artery occlusion with successful endovascular recanalization.* Neuroradiology, 2023. **65**(3): p. 619-627.
22. Mierzwa, A.T., et al., *Predictors of outcome and symptomatic intracranial hemorrhage in acute basilar artery occlusions: Analysis of the PC-SEARCH thrombectomy registry.* Eur Stroke J, 2024: p. 23969873241234713.
23. Mourand, I., et al., *Mechanical thrombectomy with the Solitaire device in acute basilar artery occlusion.* J Neurointerv Surg, 2014. **6**(3): p. 200-4.
24. Nappini, S., et al., *Bridging versus direct endovascular therapy in basilar artery occlusion.* J Neurol Neurosurg Psychiatry, 2021. **92**(9): p. 956-962.
25. Neuberger, U., et al., *Prediction of intracranial hemorrhages after mechanical thrombectomy of basilar artery occlusion.* J Neurointerv Surg, 2019. **11**(12): p. 1181-1186.
26. Ouyang, K., et al., *Posterior Circulation ASPECTS on CT Angiography Predicts Futile Recanalization of Endovascular Thrombectomy for Acute Basilar Artery Occlusion.* Front Neurol, 2022. **13**: p. 831386.
27. Pasarikovski, C.R., et al., *Outcomes of Endovascular Thrombectomy for Basilar Artery Occlusion.* Can J Neurol Sci, 2020. **47**(4): p. 479-485.
28. Pop, R., et al., *Poor clinical outcome despite successful basilar occlusion recanalization in the early time window: incidence and predictors.* J Neurointerv Surg, 2023. **15**(5): p. 415-421.
29. Ramazanoglu L, A.K.I., Gunkan A, Onal Y, Velioglu M, Topcuoglu MO, Gozke E, *The predictors of prognosis in endovascular treatment of basilar artery occlusion.* Neurology Asia, 2023. **28**(2): p. 273-282.
30. Ritvonen, J., et al., *Comatose With Basilar Artery Occlusion: Still Odds of Favorable Outcome With Recanalization Therapy.* Front Neurol, 2021. **12**: p. 665317.
31. Ryu, J.C., et al., *Delayed low cerebellar perfusion status is associated with poor outcomes in top-of-basilar occlusion treated with thrombectomy.* Front Neurol, 2023. **14**: p. 1161198.
32. Singer, O.C., et al., *Mechanical recanalization in basilar artery occlusion: the ENDOSTROKE study.* Ann Neurol, 2015. **77**(3): p. 415-24.
33. Siow, I., et al., *Bridging Thrombolysis versus Direct Mechanical Thrombectomy in Stroke Due to Basilar Artery Occlusion.* J Stroke, 2022. **24**(1): p. 128-137.
34. Son, S., et al., *Initial factors affecting the clinical outcome after successful recanalization via MR-based mechanical thrombectomy in patients with acute ischemic stroke due to basilar artery occlusion.* J Neurointerv Surg, 2016. **8**(9): p. 889-93.
35. Uno, J., et al., *Mechanical Thrombectomy for Acute Basilar Artery Occlusion in Early Therapeutic Time Window.* Cerebrovasc Dis, 2017. **44**(3-4): p. 217-224.
36. Yoon, W., et al., *Predictors of Good Outcome After Stent-Retriever Thrombectomy in Acute Basilar Artery Occlusion.* Stroke, 2015. **46**(10): p. 2972-5.
37. Abdelrady, M., et al., *Outcomes Following Mechanical Thrombectomy in Different Etiological Subtypes of Acute Basilar Artery Occlusion : Stroke Etiology and Outcome after EVT in BAO.* Clin Neuroradiol, 2023. **33**(2): p. 361-374.
38. Alexandre, A.M., et al., *Posterior Circulation Endovascular Thrombectomy for Large-Vessel Occlusion: Predictors of Favorable Clinical Outcome and Analysis of First-Pass Effect.* AJNR Am J Neuroradiol, 2021. **42**(5): p. 896-903.
39. Chen, J., et al., *Twenty-four-hour National Institute of Health Stroke Scale predicts short- and long-term outcomes of basilar artery occlusion after endovascular treatment.* Front Aging Neurosci, 2022. **14**: p. 941034.
40. Guo, M., et al., *Thrombectomy alone versus intravenous thrombolysis before thrombectomy for acute basilar artery occlusion.* J Neurointerv Surg, 2024. **16**(8): p. 794-800.
41. Hirai, S., et al., *Imaging predictors of clinical outcomes after endovascular treatment in MRI-selected patients with acute basilar artery occlusion.* Clin Neurol Neurosurg, 2023. **231**: p. 107824.
42. Huang, X., et al., *First-pass effect in patients with acute vertebrobasilar artery occlusion undergoing thrombectomy: insights from the PERSIST registry.* Ther Adv Neurol Disord, 2022. **15**: p. 17562864221139595.
43. Ishiwada, T., et al., *Influence of Bilateral Cerebellar Infarction on Functional Outcome After Endovascular Treatment for Basilar Artery Occlusion.* World Neurosurg, 2023. **171**: p. e506-e515.
44. Jiang, C., et al., *Predictors of favorable outcomes for vertebrobasilar artery occlusion after endovascular therapy within 24 hours of symptom onset.* Clin Neurol Neurosurg, 2021. **201**: p. 106422.
45. Lee, S.J., et al., *Predicting Endovascular Treatment Outcomes in Acute Vertebrobasilar Artery Occlusion: A Model to Aid Patient Selection from the ASIAN KR Registry.* Radiology, 2020. **294**(3): p. 628-637.
46. Lee, D.H., et al., *Thrombectomy in acute vertebrobasilar occlusion: a single-centre experience.* Neuroradiology, 2020. **62**(6): p. 723-731.
47. Liao, J.S., et al., *Low neutrophil-to-lymphocyte and platelet-to-lymphocyte ratios predict favorable outcomes after endovascular treatment in acute basilar artery occlusion: subgroup analysis of the BASILAR registry.* BMC Neurol, 2023. **23**(1): p. 113.
48. Liu, L., et al., *Novel Diffusion-Weighted Imaging Score Showed Good Prognostic Value for Acute Basilar Artery Occlusion Following Endovascular Treatment: The Pons-Midbrain and Thalamus Score.* Stroke, 2021. **52**(12): p. 3989-3997.
49. Maïer, B., et al., *Thrombectomy with or without Intravenous Thrombolytics in Basilar Artery Occlusion.* Ann Neurol, 2023. **94**(3): p. 596-604.
50. Nie, X., et al., *Endovascular treatment with or without intravenous alteplase for acute ischaemic stroke due to basilar artery occlusion.* Stroke Vasc Neurol, 2022. **7**(3): p. 190-199.
51. Rentzos, A., et al., *Endovascular treatment of acute ischemic stroke in the posterior circulation.* Interv Neuroradiol, 2018. **24**(4): p. 405-411.
52. Sang, H.F., et al., *Mechanical Thrombectomy Using Solitaire in Acute Ischemic Stroke Patients with Vertebrobasilar Occlusion: A Prospective Observational Study.* World Neurosurg, 2019. **128**: p. e355-e361.
53. Sun, X., et al., *Endovascular treatment for acute basilar artery occlusion: a single center retrospective observational study.* BMC Neurol, 2019. **19**(1): p. 315.
54. Sun, D., et al., *Outcome prediction value of critical area perfusion score for acute basilar artery occlusion.* Interv Neuroradiol, 2023. **29**(6): p. 702-709.
55. Sun, D., et al., *Predictors of poor outcome after endovascular treatment for acute vertebrobasilar occlusion: data from ANGEL-ACT registry.* Neuroradiology, 2023. **65**(1): p. 177-184.
56. Werner, M., et al., *Mechanical thrombectomy in acute basilar artery occlusion: A safety and efficacy single centre study.* Interv Neuroradiol, 2016. **22**(3): p. 310-7.
57. Wu, L., et al., *Long-term outcome of endovascular therapy for acute basilar artery occlusion.* J Cereb Blood Flow Metab, 2021. **41**(6): p. 1210-1218.
58. Wu, M., et al., *Predictors of mortality for acute vertebrobasilar artery occlusion receiving endovascular treatment.* Acta Neurol Scand, 2021. **144**(4): p. 433-439.
59. Zhang, X., et al., *Predictors of Good Outcome After Endovascular Treatment for Patients with Vertebrobasilar Artery Occlusion due to Intracranial Atherosclerotic Stenosis.* Clin Neuroradiol, 2019. **29**(4): p. 693-700.
